# Mechanisms underlying familial aggregation of exceptional health and survival: A three‐generation cohort study

**DOI:** 10.1111/acel.13228

**Published:** 2020-09-04

**Authors:** Kaare Christensen, Mary K. Wojczynski, Jacob K. Pedersen, Lisbeth A. Larsen, Susanne Kløjgaard, Axel Skytthe, Matt McGue, James W. Vaupel, Michael A. Province

**Affiliations:** ^1^ Department of Public Health Danish Aging Research Center University of Southern Denmark Odense Denmark; ^2^ Department of Genetics Washington University School of Medicine St. Louis MO USA; ^3^ Department of Psychology University of Minnesota Minneapolis MN USA; ^4^ Center on Population Dynamics University of Southern Denmark Odense Denmark

**Keywords:** family study, healthy aging, longevity, multi‐generation study

## Abstract

The familial resemblance in length of adult life is very modest. Studies of parent‐offspring and twins suggest that exceptional health and survival have a stronger genetic component than lifespan generally. To shed light on the underlying mechanisms, we collected information on Danish long‐lived siblings (born 1886–1938) from 659 families, their 5379 offspring (born 1917–1982), and 10,398 grandchildren (born 1950–2010) and matched background population controls through the Danish 1916 Census, the Civil Registration System, the National Patient Register, and the Register of Causes of Death. Comparison with the background, population revealed consistently lower occurrence of almost all disease groups and causes of death in the offspring and the grandchildren. The expected incidence of hospitalization for mental and behavioral disorders was reduced by half in the offspring (hazard ratio 0.53, 95% confidence interval 0.45–0.62) and by one‐third in the grandchildren (0.69, 0.61–0.78), while the numbers for tobacco‐related cancer were 0.60 (0.51–0.70) and 0.71 (0.48–1.05), respectively. Within‐family analyses showed a general, as opposed to specific, lowering of disease risk. Early parenthood and divorce were markedly less frequent in the longevity‐enriched families, while economic and educational differences were small to moderate. The longevity‐enriched families in this study have a general health advantage spanning three generations. The particularly low occurrence of mental and behavioral disorders and tobacco‐related cancers together with indicators of family stability and only modest socioeconomic advantage implicate behavior as a key mechanism underlying familial aggregation of exceptional health and survival.

## Graphical abstract

This Danish three‐generation cohort study of more than 15,000 descendants of long‐lived siblings revealed consistently lower occurrence of almost all disease groups and causes of death in the offspring and grandchildren—most pronounced for mental and behavioral disorders and tobacco‐related cancers. The longevity‐enriched families have a general health advantage that transmits across multiple generations, and the disease and demographic characteristics implicate behavior as a key mechanism underlying the familial aggregation of exceptional health and survival.

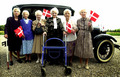

## 1 INTRODUCTION

Several complex diseases and conditions show familial aggregation over three generations. In the Framingham study, early‐onset hypertension in the first generation was associated with hypertension in the grandchildren generation even after adjusting for parental early‐onset hypertension and lifestyle factors (Niiranen et al., 2017). Similarly, coronary heart disease, birth weight, body mass index, and depression have been found to track over three generations (Emanuel, Filakti, Alberman, & Evans, 1992; Josefsson, Vikström, Bladh, & Sydsjö, 2019; Murrin, Kelly, Tremblay, & Kelleher, 2012; Ranthe et al., 2015; Weissman et al., 2016). In the Utah Population Database, multiple cancers were determined to have a familial component: transmission persisted up to fifth‐degree relatives (Kerber & O'Brien, 2005).

**FIGURE 1:**
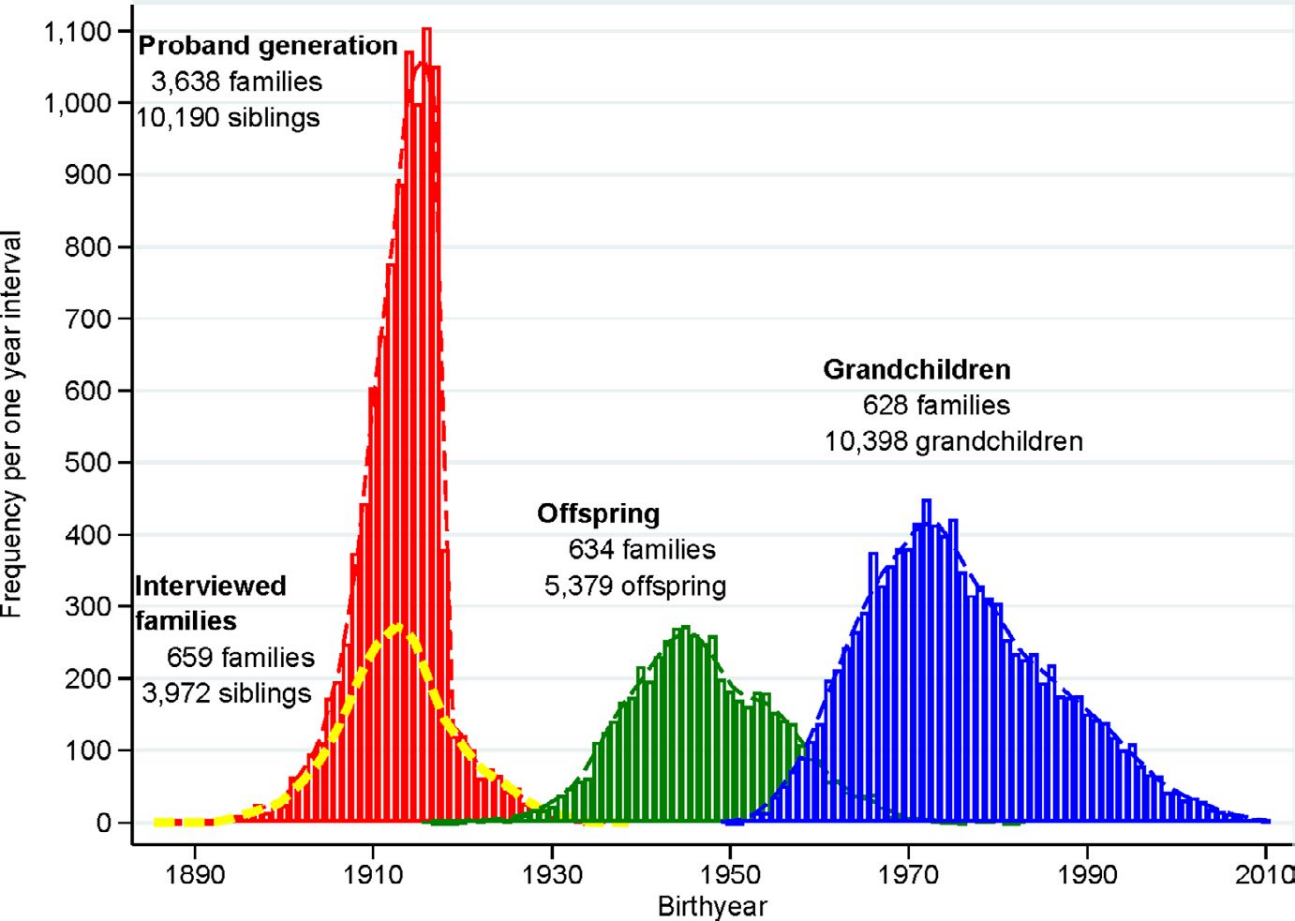
Birth year distribution in the three generations of LEF families: the proband generation, the generation of the offspring of interviewed families, and the grandchild generation

**FIGURE 2:**
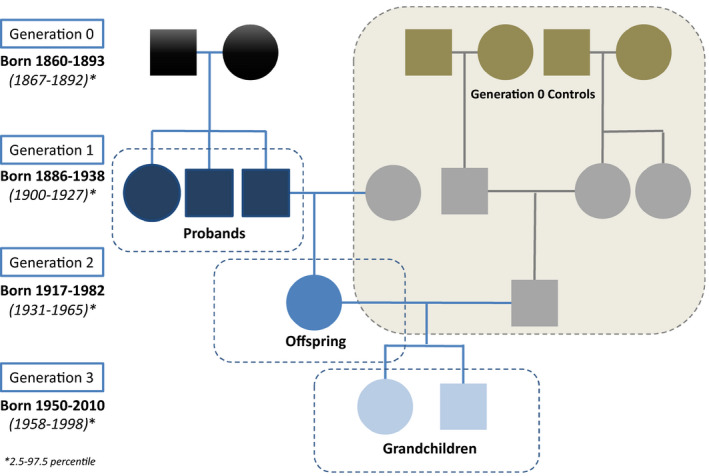
Pedigree structure for the longevity‐enriched families and control families. The blue figures in Generation 1 are the long‐lived siblings who are the probands in the study. For simplicity, only one of the offspring of the probands are shown in the pedigree. The green/gray figures are married‐in control families in the 1916 Census analyses of the socioeconomic conditions for Generation 0 in the longevity‐enriched families. For each of the offspring and grandchildren, 10 age‐ and sex‐matched controls were randomly selected from a 5% random sample of the Danish population through register linkage (not shown in the figure)

Several studies have shown that longer parental lifespan decreases the incidence of specific diseases in the offspring (Florez et al., 2011; Lipton et al., 2010). The *Long Life Family Study* and the *Leiden Longevity Study* demonstrated many health advantages in children of long‐lived sibships (Ash et al., 2015; Barral et al., 2012, 2017; Cosentino et al., 2013; Dekker et al., 2009; Newman et al., 2011; Rozing et al., 2010; Stijntjes et al., 2013; Westendorp et al., 2009; Wijsman et al., 2010), but it is less clear to what degree exceptional health advantages track over multiple generations. Demographic studies indicate that multi‐generational correlation of lifespans is very modest. Recent research has further suggested that variation in adult lifespan is even less heritable than previously estimated (Kaplanis et al., 2018) and that assortative mating (genetic and/or environmental) contributes substantially to the very modest familial resemblance in length of adult life (Ruby et al., 2018). However, previous research also suggests that, for unknown reasons, an exceptionally long life has stronger genetic associations than lifespan generally (van den Berg, Beekman, Smith, Janssens, & Slagboom, 2017; van den Berg et al., 2019). An intergenerational tracking of exceptional health and survival could be due to low risk of specific diseases or a fundamentally slower aging process that results in a non‐specific lowering of risk for diseases in general. Getting a better understanding of the determinants of exceptionally healthy aging can potentially be of great public health importance in an aging society. Here, mortality and disease occurrence in a large sample of first‐ and second‐generation descendants of Danes from exceptionally long‐lived sibships are compared to an unselected sample of Danes (Figures 1 and 2). Our analyses seek to determine whether the health benefits of a family history of exceptional longevity: (a) transmit across multiple generations; (b) are associated with a general, as opposed to specific, lowering of disease risk, and (c) are associated with social indicators.

## 2 RESULTS

### 2.1 Offspring: Disease incidence

**FIGURE 3:**
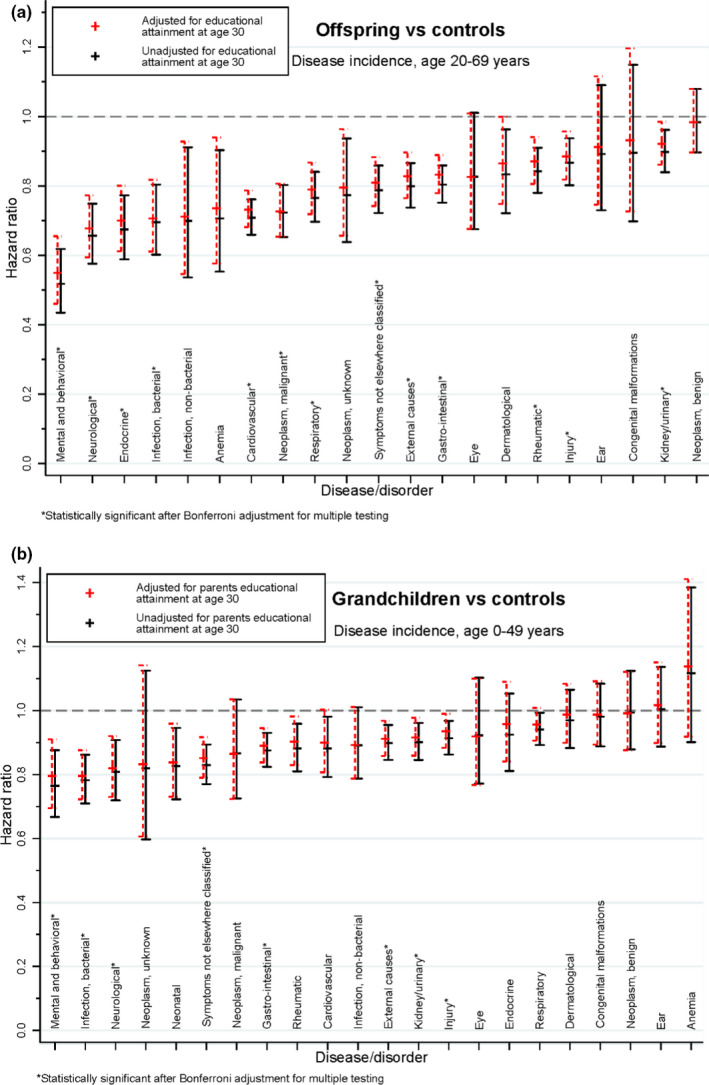
(a) Offspring of Danish longevity‐enriched families vs. age‐ and sex‐matched controls from a random sample of the general Danish population: comparison of incidence of disease‐specific hospitalization for 22 major disease categories. Bars indicate 95% CI. (b) Grandchildren of Danish longevity‐enriched families vs. age‐ and sex‐matched controls from a random sample of the general Danish population: comparison of incidence of disease‐specific hospitalization for 22 major disease categories. Bars indicate 95% CI.

The disease incidence estimates were lower among offspring of long‐lived siblings compared to population controls in 21 disease categories, while the 22nd had too few events to allow assessment (Figure 3 and Table S4). The corresponding hazard ratios (HRs) comprised one HR at 0.52 (mental and behavioral disorders), two at 0.60–0.69, eight at 0.70–0.79, nine at 0.80–0.90, and one at 0.98 (neoplasms, benign). The number for tobacco‐related cancer was 0.60 (0.51–0.70) and for lung cancer alone 0.34 (0.24–0.49). Of the 22 categories, incidence was significantly lower among 17 disease categories (13 when adjusted for multiple testing using the conservative Bonferroni correction). Sex‐stratified results were similar and are given in Table S4 in Appendix S1. Adjustment for attained educational level at age 30 resulted in only small changes in the HRs, in most cases toward HR 1 (Figure 3, Figures S3 and S7).

### 2.2 Offspring: Cause‐specific mortality

**FIGURE 4:**
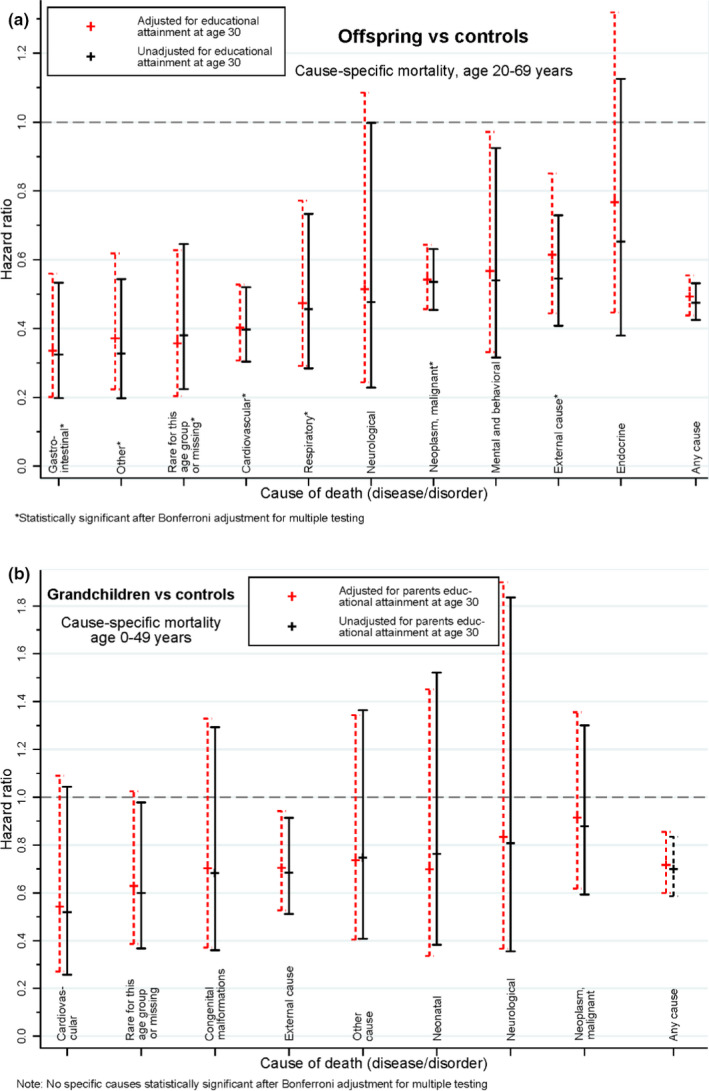
(a) Offspring of Danish longevity‐enriched families vs. age‐ and sex‐matched controls from a random sample of the general Danish population: Comparison of cause‐specific mortality for 10 cause‐of‐death categories. Bars indicate 95% CI. (b) Grandchildren of Danish longevity‐enriched families vs. age‐ and sex‐matched controls from a random sample of the general Danish population: Comparison of cause‐specific mortality for eight cause‐of‐death categories. Bars indicate 95% CI.

The all‐cause mortality for the offspring was half the expected based on the control population (HR = 0.48, 95% confidence interval 0.42–0.53). For the 10 specific causes of death considered (Figure 4 and Table S5), the HRs comparing offspring to controls ranged from 0.32 to 0.65, and, except for the category of endocrine, metabolic or nutritional diseases (HR = 0.65), all 10 HRs were significantly below 1 (7 HRs with the Bonferroni correction). (Appendix S1 and Table S2).

### 2.3 Grandchildren: Disease incidence

For 20 of the 22 disease categories, the HRs were below 1 (Figure 3, Table S6 and Figure S3), though less markedly so than among the offspring generation: two were below 0.79 as were tobacco‐related cancers with 0.71 (0.48–1.05), nine at 0.80–0.89, nine at 0.90–0.99, one at 1.00, and one at 1.12. For 12 categories, the HRs were significantly below 1 (8 categories with Bonferroni correction).

### 2.4 Grandchildren: Cause‐specific mortality

For all‐cause mortality, the HR was 0.70 (95% confidence interval 0.59–0.84) and the survival advantage included the infant period (0–365 days) with a total mortality of 0.34% in the grandchildren and 0.54% in the control group (*p* = 0.045). For the eight specific causes of death considered (Figure 4 and Table S7), the HRs comparing grandchildren to controls ranged from 0.52 to 0.88. With a Bonferroni correction for eight comparisons, none of the differences remained statistically significant.

### 2.5 Intergenerational similarity in disease occurrence

**FIGURE 5:**
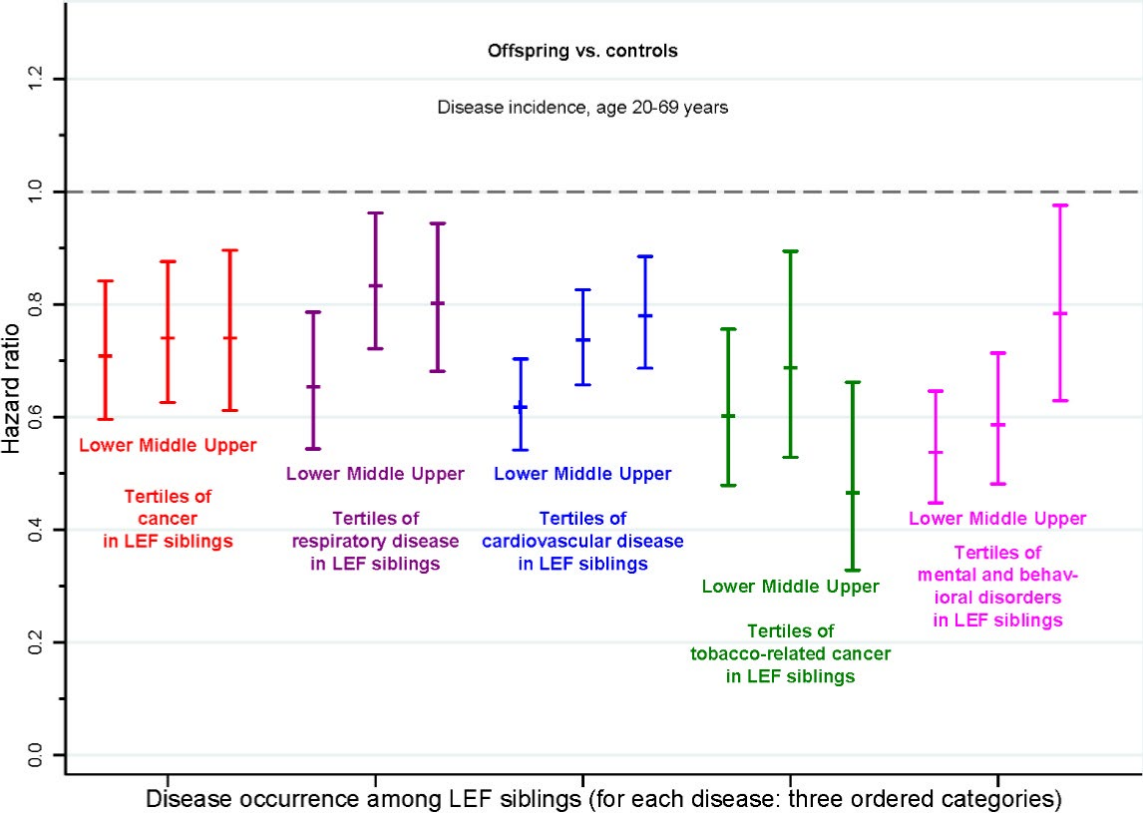
Trans‐generational similarity in disease occurrence in Danish longevity‐enriched families. For each of the five major disease categories, the longevity‐enriched families were divided into three tertiles (lower, middle, and upper) based on the incidence of hospitalization for the specific disease category among siblings from each family in the proband generation. The tertile subgroup variable was then related to the incidence of hospitalization for the same disease category in the offspring sample compared to background population controls. Bars indicate 95% CI. The figure suggests that the longevity‐enriched families are not comprised of families that avoid specific disease categories (e.g., “cancer‐avoiding families”) but instead of families that have a general, as opposed to specific, lowering of disease risk

For all five major types of disease history in the longevity‐enriched families (LEF) proband generation, the disease incidence in the offspring was well below that in the controls, regardless of whether the offspring belonged to the lower, middle, or upper tertile of LEF families' disease history. Point estimates of HRs ranged from 0.47 to 0.83, and for all diseases and tertiles, incidence was significantly lower among the offspring (Figure 5 and Tables S8 and S9). For both cardiovascular diseases and mental and behavioral disorders, there was a significant difference in the HRs between families with the lowest, middle, and highest extent of familial disease history. For both these disease categories, there was a small trend toward relatively lower disease incidence among offspring of families from the lowest risk tertile. Nonetheless, even for these categories, offspring from the highest risk tertile still had lower disease risk than the population background. For diseases of the respiratory system, cancer overall, and tobacco‐related cancer, however, there was no significant difference between the three tertiles. In the sex‐specific strata, the results were similar (Table S9). Hence, these within‐family analyses showed a general, as opposed to specific, lowering of disease risk in the offspring of long‐lived siblings.

### 2.6 Educational attainment in the offspring generation

The differences in educational attainment between offspring and controls were small to moderate and most pronounced in the female population (Figure S6), and they only changed the estimates marginally (Figures 3 and 4).

### 2.7 Marriage, divorce, and age at first child

The frequency of teenage marriage was markedly lower than expected in the offspring and grandchildren of long‐lived siblings: 0.58 (95% confidence interval 0.52–0.66) and 0.64 (95% confidence interval 0.48–0.86), respectively (Results in Appendix S1 and Figures S8 and S10). Similarly, the frequency of teenage parenthood in offspring was also about half the expected: 0.43 (95% confidence interval 0.38–0.50). The distribution of the number of children of each offspring was similar to that of the controls (Figures S12 and S13). Divorce rates within the first five years after marriage were markedly lower among offspring (HR = 0.55 (95% confidence interval 0.46–0.65)) and moderately lower among grandchildren: 0.76 (95% confidence interval: 0.68–0.85) than among their respective, matched controls (Figures S9 and S11).

### 2.8 Long‐lived siblings: Childhood socioeconomic conditions (The 1916 Census)

The proband control sample consisted of 358 families after implementing the restriction criteria of at least two children born in the same parish in Denmark before 1 April 1918 (G0 generation in Figure 2). Occupational information could be found in the 1916 Census for 99% of the G0 individuals, while the other wealth information was registered for approximately 50%–80% of the G0 individuals (Table S10). No statistically significant difference in income, wealth, or taxes was found between the LEF sample and the control sample, but for all these measures of economic status, a higher mean was observed in the control sample (Table S10). The occupation distribution of the G0 was similar in the LEF and the control sample except for the small group of “non‐material occupations” (professional occupations) (7.1% vs. 3.1%, *p* < 0.01) which mainly consisted of public servants and teachers. Most of the G0 men had either agricultural, crafts, or industrial occupations (Figure S14). Compared to their controls, the long‐lived siblings' parents lived longer: 4.1 years (95% confidence interval 2.4–5.9) for fathers and 4.7 years (95% confidence interval 2.6–6.7) for mothers.

## 3 DISCUSSION

Having a long‐lived parent or grandparent who had at least one long‐lived sibling is associated with a substantial health and survival advantage in our study indicating that the health benefits of a family history of exceptional longevity transmit across multiple generations. Most notable is the strength of the associations, and that these are found for a wide range of diseases and causes of death, suggesting a fundamentally slower aging in these families and not just avoidance of specific diseases. The combination of a particularly low incidence of mental and behavioral disorders and tobacco‐related cancers combined with demographic characteristics such as low occurrence of teenage parenthood and early marriage and divorce implicate behavior as a key mechanism underlying the three‐generation health and survival advantage observed. We found no evidence that the associations were driven by socioeconomic advantages in the longevity‐enriched families either in the 1916 census or in the civil registration system over the last half‐century.

Behavior is influenced by a complex set of bio‐psycho‐social and cultural factors. Risk‐taking and personality traits like extraversion, agreeableness, and conscientiousness as well as behaviors such as smoking are known to have substantial genetic contribution to their variation in very diverse populations (Bouchard & McGue, 2003; Saccone et al., 2007; Wang, Zheng, Xuan, Chen, & Li, 2016) and, moreover, to be associated with survival (Jokela et al., 2013; Roberts, Kuncel, Shiner, Caspi, & Goldberg, 2007). Similarly, childhood rearing environment including role models is associated with later adult behaviors (Kendler, Ohlsson, Sundquist, & Sundquist, 2018). The long‐lived siblings in our study were from large sibships: average 7.2 siblings compared to 6.6 in the 358 control families that were selected among families with at least two children. Hence, the long‐lived siblings tended to come from rearing environments unaffected by early parental death or divorce. We were able to demonstrate that this tracked over generations with lower early adult life mortality and divorce rates also in the offspring and grandchildren generation. The familial stability characteristics are also in line with the remarkably low occurrence of hospitalizations for mental and behavioral disorders observed both in the offspring and the grandchildren generation—disorders that are strongly associated with adverse socioeconomic conditions, health behaviors, and survival (Brink et al., 2018). Our study of personality among offspring of long‐lived siblings also found a profile that tends to be associated with good health: low score in neuroticism, high in extraversion, and in the high‐average range in conscientiousness and agreeableness (Andersen et al., 2013).

The proband siblings were selected based on their exceptional survival. Nationwide Danish register data have previously shown that non‐agenarians and centenarians have been hospitalized substantially less earlier in life than their shorter‐lived contemporaries, indicating that exceptional survival is associated with healthy aging earlier in life (Engberg, Oksuzyan, Jeune, Vaupel, & Christensen, 2009).

Among the strengths of the study is that the cohort information for the children and grandchildren as well as their controls are obtained through national registers and hence not dependent on participation in a survey. This minimizes loss to follow‐up and healthy participant bias at the expense of no available survey data on behavior. Using the Danish Cancer Registry, we have demonstrated low occurrence of cancers in the offspring of long‐lived siblings (Pedersen et al., 2015). We confirmed these findings using the Danish Patient Registry, and we extended the analyses to the grandchildren who, despite being followed only up to age 49, also showed lower cancer rates compared to controls, most pronounced for tobacco‐related cancers, although only borderline statistically significant. The lower rates of tobacco‐related cancers in offspring and grandchildren could be due to both lower smoking frequency and/or higher biological resilience to the carcinogenic effects of tobacco in individuals with a familial history of longevity. Smoking habits in offspring were available for the subset of families in our study who also participated in the *Long Life Family Study*, and Pedersen et al. (2015) showed that the prevalence of smokers in the offspring was 24% lower than that predicted from age‐ and sex‐specific smoking prevalence in the Danish population (Den Nationale & Sundhedsprofil, 2010).

The Danish national healthcare system with no out‐of‐pocket costs for hospitalizations further ensures nearly complete registration of the hospitalizations in the cohorts independent of socioeconomic status. On the other hand, the Scandinavian welfare state model may limit the external validity of our findings, and these may not replicate in less egalitarian countries.

Socioeconomic position could be expected to be an underlying factor in familial aggregation of exceptional health and survival as a social gradient in health and mortality is present in contemporary Denmark (Brønnum‐Hansen, Eriksen, Andersen‐Ranberg, & Jeune, 2017). However, pronounced differences in working‐age mortality appeared only in the post‐world war II period in a micro‐level study of adult mortality in Southern Sweden (Bengtsson & Dribe, 2011). In the present study, we did not find evidence that socioeconomic status was an important factor in the familial aggregation of exceptional health and survival.

The 1916 census data showed no systematic differences between the childhood socioeconomic conditions for the long‐lived siblings compared to offspring‐spouse control families. Furthermore, the educational differences between the offspring and the background population were small for males and moderate for females, and controlling for these differences in the analyses of disease incidence and cause‐specific mortality only changed the estimates marginally and generally in the expected direction. However, we cannot rule out some residual socioeconomic confounding not captured by education.

The survival advantage tracking over the three generations is remarkable in size and consistency and across multiple distinct common causes of death. This is in line with a study by O'Brien et al. (2007) based on the Utah population database showing that individuals from longer‐lived families have lower mortality from most age‐related diseases. Similarly, for health measured as disease incidence, we found a strong tracking over three generations, suggesting that these longevity‐enriched families have a general health advantage. Pathology increases with age in these families, though to a lesser degree than in the controls indicating a fundamentally slower aging. Studies of multi‐generation longevity‐enriched families with better health and survival at all ages can deepen our understanding of the diversity of the fundamental physiological processes of aging, which can potentially have a major public health impact in an aging world.

It is challenging to disentangle the effects of genetic factors and shared family environment on longevity in traditional family studies. However, twin studies from Scandinavia have suggested that about a quarter of the variation in adult lifespan can be attributed to genetic differences between individuals and that the heritability is likely to increase at higher ages and to be strongest for exceptional survival (Hjelmborg et al., 2006; Ljungquist, Berg, Lanke, McClearn, & Pedersen, 1998). However, few genetic variants associated with longevity have been identified (Deelen et al., 2019), potentially because of rare variants and “phenocopies,” that is, individuals that live long by chance. Using Dutch historical data, van der Berg et al. (2020) recently showed that longevity is transmitted for at least two subsequent generations only when at least 20% of all relatives are long‐lived. This is in line with our results and suggests that longevity‐enriched families can be a powerful study sample for facilitating the discovery of novel genetic variants promoting exceptional health and survival—not only rare variants unique to the family but also common variants.

The underlying molecular mechanisms for better health and survival across three generations could include interactions between genetic variants (private or common) and epigenetic factors (multi‐generational or trans‐generational) interacting with shared behavioral, social, and cultural factors (Miska & Ferguson‐Smith, 2016; Young, Benonisdottir, Przeworski, & Kong, 2019). Most likely, all these factors are intertwined; for example, parental stability and conscientiousness are influenced by genetic variants that are transmitted to the next generation and correlated with the rearing environment that may again affect epigenetics both trans‐ and multi‐generational. Overall, our study indicates that behavior, which has a genetic component, is a key mechanism in the familial aggregation of exceptional health and survival observed in Denmark in the last century.

## 4 METHODS

### 4.1 Study population

The study population comprised three generations of members of Danish longevity‐enriched families (LEFs) along with matched population controls for the latter two generations. LEFs were defined as families where at least two siblings had attained an age ≥88 years and were still alive at the time of recruitment, which took place between 2006 and 2009. In our study population, 99.5% of the recruited families included at least two siblings who survived to age 90 years, whereas for the remaining families, one sibling survived well past 90 years and at least one other sibling survived to age 89 years. In the supplement method, we provide more details. The LEFs included 3,972 siblings from 659 long‐lived sibships (including the 76 Danish families in the *Long Life Family Study* (Ash et al., 2015; Barral et al., 2012, 2017; Cosentino et al., 2013; Newman et al., 2011), their 5379 offspring, and their 10,398 grandchildren (Figures 1 and 2, Table S1A,B) (Pedersen et al., 2015; Sebastiani et al., 2009).

For comparison, controls were selected from a 5% random sample of the Danish population. For each offspring, ten controls were selected matched on sex and birth year but otherwise randomly selected. For each grandchild, ten controls were selected so that they matched on sex and birth year and so that the birth year of the grandchild's LEF parent matched the birth year of the parent of the control with the same sex as the LEF parent. Since the definition of a LEF implied that its members, to a larger extent than that which is the case in the general population, were born in Denmark, both LEF families and the controls were restricted to individuals born in Denmark.

### 4.2 Disease incidence

Information on disease in LEF offspring, grandchildren, and their respective control groups was obtained from the Danish National Patient Register that has been in operation since 1977 (Lynge, Sandegaard, & Rebolj, 2011). In the following, we use the term *disease incidence* as synonymous with disease‐specific inpatient hospitalization using the main diagnosis of each hospitalization only. The disease incidence was studied for the period 1977–2011 in 22 categories based on the main groupings in the ICD‐10 classification of diseases. Only hospitalizations of inpatients were used, and for each disease category, only the first occurrence for each study participant within the study period was included; later occurrences were not included. Moreover, only the main discharge diagnosis for each hospitalization was considered. As each disease category was analyzed separately, the occurrence of disease in a given category did not preclude occurrences in the other categories. For the estimation of disease incidence ratios, the study population of the offspring generation was restricted to ages 20–69 years and the grandchild generation to ages 0–49 years. There was very little observational time outside these age ranges (Methods in Appendix S1, Tables S2 and S3, and Figure S2).

### 4.3 Cause‐specific mortality

The same age period restrictions were implemented in the study of cause‐specific mortality. Causes of death data were available from the Danish Register of Causes of Death (Helweg‐Larsen, 2011) for the period 1970–2010, although for the grandchild population, the information was only available after 1973 (Methods in Appendix S1 and Table S2).

### 4.4 Intergenerational similarity in disease occurrence

To determine whether a family history of exceptional longevity provided general protection against diseases, we focused on trans‐generational transmission of risk for five major disease categories: cardiovascular, respiratory, cancer, tobacco‐related cancer, and mental and behavioral disorders (see Methods in Appendix S1 and Table S2). For each disease category, LEF families were divided into three tertiles (lower, middle, and upper) based on the incidence of the specific disease category among siblings from each family in the proband generation. The tertile subgroup variable was then related to risk in the offspring sample, separately for each of the five categories. A general protection against diseases would be supported if we observed lower disease category risk in offspring regardless of their family's tertile classification, as this would indicate that what was important in predicting low disease incidence was a family history of long life and not a family history of avoiding the specific disease.

### 4.5 Adjustment for educational attainment of the offspring generation

To assess the possible impact of the difference in educational level in the offspring generation on any disease disparity between the LEF offspring and grandchildren and their respective controls, the comparisons of disease incidence and cause‐specific mortality were adjusted for the attained educational level at age 30 obtained by linking to the Danish Population's Education Register (for details see Methods in Appendix S1).

### 4.6 Offspring and grandchildren: Marriage, divorce, and age at first child

For individuals in Denmark alive in 1968 or later, marital statuses from 1968 up to 2013, as well as start date and end date (if applicable) of status, were available from the Danish Civil Registration System (Pedersen, Gøtzsche, Møller, & Mortensen, 2006) (for details see Methods in Appendix S1).

### 4.7 Long‐lived siblings: Childhood socioeconomic conditions (The 1916 Census)

To assess the possible impact of socioeconomic conditions in childhood for the long‐lived siblings, information on income and occupation for parents of 645 of the 659 sibships (Generation 0 in Figure 2) was identified in the 1916 census in Denmark. The control groups comprised of the “corresponding” grandparents to the spouses to offspring of the long‐lived siblings (Generation 0 in Figure 2). Only Generation 0 controls who had two children born in the same parish in Denmark before 1 April 1918 were included as this was the study base from which the LEF families were identified (Pedersen et al., 2015). Occupation distribution was grouped into four main groups (Statistics Denmark, 1920).

### 4.8 Statistical analysis

Incidence and mortality ratios were estimated using Cox regression, stratified on matching criteria: birth year and sex, and with age as time variable. Moreover, robust standard errors were used to adjust for family clustering in the LEF families of the study population. Time to first marriage and first divorce was displayed using Kaplan–Meier survival estimates.

Proportionality of hazards over the entire age ranges was assessed by graphing the Kaplan–Meier log‐cumulative hazard functions against age/time for the LEF group and the control group. When the proportional hazards assumption was not met over the entire age/time range, HRs were provided for sub‐intervals for which the proportionality assumption was not violated.

Comparison of income in Generation 0 between LEF families and control families was done using unpaired two‐sample *t* test with unequal variances and with the Mann–Whitney non‐parametric test. Stata, version 16.0, was used for all statistical analyses.

## CONFLICT OF INTEREST

No conflict of interest for any of the authors.

## AUTHOR CONTRIBUTIONS

KC, MKW, and MAP developed the concept and designed the study. JKP, LAL, SK, and AS did the data acquisition and the data analysis. KC, MKW, and JKP drafted the report, and all authors critically revised the report for intellectual content and gave their final approval of the version to be published.

## ETHICAL APPROVAL

The study has been approved by The Regional Scientific Ethical Committees for Southern Denmark (S‐VF‐20030227), The Danish Data Protection Agency (J.nr. 2008‐41‐1753), and University of Southern Denmark, Research & Innovation Organisation (J.nr. 10 931). The participants gave informed consent before taking part in the in‐person studies.

## Supporting information

Appendix S1

## Data Availability

Data cannot be made available for legal reasons. We have obtained the data through a government agency (Statistics Denmark) that, due to confidentiality and privacy concerns, has restriction on data use. Among those are that researchers cannot share data with other researchers and that the data can only be used for the specific project for which they are requested. However, the code used for data preparation and/or analyses can be made available upon request.
