# Supplementary material for: Mechanisms underlying familial aggregation of exceptional health and survival: A three‐generation cohort study
Source: Aging Cell. 2020 Sep 4;19(10):e13228. doi: 10.1111/acel.13228 (PMC7576291; doi:10.1111/acel.13228)
Supplement: Supplementary file 1 — Appendix S1 [file ACEL-19-e13228-s001.docx]

**Supplement – methods and results**

**Table of contents**

Methods………………………………………………………………………………………. 1

Results…………………………………………………………………………………………3

References……………………………………………………………………………………. 6

Supplement tables……………………………………………………………………………. 7

Supplement figures…………………………………………………………………………...24

**Methods**

*Study population*

Longevity-enriched families (LEFs) were defined as families where at least two siblings had attained an age ≥ 88 years and were still alive at the time of recruitment, which took place between 2006 and 2009. In our study population, 99.5% of the recruited families included at least two siblings who survived to age 90 years, whereas for the remaining families, one sibling survived well past 90 years and at least one other sibling survived to age 89 years. The initial LEFs was carried out by identifying individuals satisfying the age criteria in a population-based register of all individuals alive and living in Denmark after 1968 (the Danish Civil Registration System; (Pedersen *et al*, 2006; Pedersen, 2011)), and matching these on surnames and birth parishes. From these matches, siblings were verified through church records from which their parents’ identities were available. These siblings comprised the proband generation of LEFs. Over the recruitment period, LEF probands were invited to participate in structured interviews during which information on the identity of other siblings as well as the identities of their own and that of their siblings’ offspring were obtained (names and birth dates). Using this information to uniquely identify the offspring generation in the Danish Civil Registration System (Pedersen, 2011; Pedersen *et al*, 2006), further links to their offspring (i.e. the LEF grandchildren) were available in the registers, resulting in the three generations of LEF families in the study population (Supplement Figure S1).

For comparison, controls were selected from a 5% random sample of the Danish population. For each offspring, ten controls were selected matched on sex and birth year, but otherwise randomly selected. For each grandchild, ten controls were selected so that they matched on sex and birth year and so that the birth year of the grandchild’s LEF parent matched the birth year of the parent of the control with the same sex as the LEF parent. Since the definition of a LEF implied that its members, to a larger extent than that which is the case in the general population, were born in Denmark, both LEF families and the controls were restricted to individuals born in Denmark.

In Tables S1A and S1B we have provided various characteristics of LEF siblings, offspring, controls, and the control groups of offspring and grandchildren.

All data linkages were done within Statistics Denmark.

*Disease incidence*

Information on disease incidence, that is, disease-specific inpatient hospitalization was obtained from the Danish National Patient Registry (Schmidt *et al*, 2015), which at its onset in 1977 covered inpatients in somatic ward but was gradually expanded. From 1995 onwards it included all outpatient activities, emergency room contacts, and psychiatric inpatients activities. While it is considered virtually complete in its coverage of hospitalizations (Lynge *et al*, 2011; Schmidt *et al*, 2015), the validity of specific diagnosis varies. A review study found that compared to various reference standards the positive predictive value of 139 disease categories (at three to five digits level of ICD-10 code) varied from <15% to 100% (Schmidt *et al*, 2015). However, when considering only categories with ICD-10 code with three digits, the major part of categories had positive predictive values between 80% and 100%. The disease incidence was studied for the period 1977-2011 in 22 categories based on the main groupings in the ICD-10 classification of diseases. For the period 1977-1993, when diseases in Denmark were classified according to ICD-8, the corresponding codes were selected by allocating ICD-8 sub-categories to the ICD-10 main grouping that was judged the best match. Conversion tables from the Swedish National Board of Health and Welfare were used to check these matches^15,16^ (Supplemental Tables S2 and S3 and supplemental Figure S2).

For the analyses of the 22 main disease groupings, only hospitalisations of inpatients were used, and for each disease category, only the first occurrence for each study participant within the study period was included; later occurrences were not included. Moreover, only the main discharge diagnosis for each hospitalisation was considered. As each disease category was analysed separately, the occurrence of disease in a given category did not preclude occurrences in the other categories.

*Cause-specific mortality*

Causes of death data were available from the Danish Register of Causes of Death^17^ for the period 1970-2010, although for the grandchild population, the information was only available after 1973. The reporting of cause of death to the register is based on information recorded on death certificates, and no central/regular validation is performed, and misclassification is an issue especially for ill-defined diseases. For the offspring and grandchild study populations, cause of death was initially classified into the 22 disease categories described above with two added categories for “Other causes” and “Missing causes”. Subsequently, the categories with low frequencies were combined into one “Rare causes in the age group” category reducing the 24 categories to 11 in the case of the offspring generation and to nine categories in the case of the grandchild generation (Supplemental Table S2). The same age period restrictions as for disease incidence were implemented in the study of cause-specific mortality.

*Intergenerational similarity in disease occurrence*

For the intergenerational analyses, the cancer category comprised all but non-melanoma skin cancer. Tobacco-related cancer comprised cancer sites/types classified as such according to the American Cancer Society (American Cancer Society, 2019): lip, tongue, mouth, salivary glands, pharynx, esophagus, stomach, colon, rectum and anus, pancreas, nose and sinuses, larynx, lung, cervis uteri, ovary and other female genital organs, kidney, bladder and other/unspecified urinary organs, and acute leukemia. For mental and behavioural disorders, all hospital contacts and both primary and secondary diagnoses were included in order to enhance the disease sensitivity for this category. For each disease, only the first diagnosis of each sibling in the 35-year period from 1977 to 2011 was included in the calculation of the family disease rate in the sibling generation.

*Educational attainment of the offspring generation*

The differences in educational attainment between offspring and controls were small to moderate and most pronounced in the female population (Figure S6). Among males, 5.5% more among the controls had either a missing value of or primary/lower secondary education as highest attained educational level compared to male offspring (35.4% vs. 29.9%), 1.1% more among controls had upper/post-secondary, non-tertiary education (46·0% vs. 44·9%), and 6.6% less among controls (18.6% vs. 25.2%) had tertiary education (short cycle/bachelor’s/master’s or doctoral) as highest attained educational level. Among females (controls vs. offspring), the differences in proportion with the same level of highest attained educational level were 11.4% (46.7% vs. 35.3%) for primary/lower secondary education, -1.3% (34.4% vs. 35.7%) for upper/post-secondary, non-tertiary, and -10.1% (18.9% vs. 29.0%) for tertiary education.

The information on educational attainment at age 30 was obtained by linking to the Danish Population’s Education Register (Jensen and Rasmussen, 2011). Educational attainment was available for the years 1980-2011 with status of educational attainment on October 1 of each year, so that for individuals born after October 1, 1949, and before October 1, 1981, there was information on educational attainment at age 30. The eight individuals (0.02%) who were born after October 1, 1981, were all born before the end of 1982, and were assigned their educational attainment on October 1, 2011 (i.e. around age 29 years). For the 3,372 (63.2%) offspring and 29,798 (65.5%) controls born before October 1, 1949, the status of educational attainment on October 1, 1980, was used. Since 40 (0.8%) offspring died and 53 (1.0%) had emigrated/disappeared before year of educational link, and, similarly, 634 (1.4%) controls died and 612 (1.3%) had emigrated/disappeared before this year, the study population for educational attainment comprised 5,240 offspring and 44,232 controls. In the linkage procedure, 88 (1.7%) of the 5,240 offspring and 829 (1.9%) of the 44,232 controls were missing from the educational register, and a further 59 (1.1%) offspring and 559 (1.3%) controls had missing values of the variable of educational attainment. For the 3,317 offspring and 28,986 controls in the study population, status of highest educational attainment was obtained via register linkage in Statistic Denmark. The age at which the highest educational attainment was obtained was below age 30 for 2,136 (64.4%) offspring and 18,631 (64.3%) controls, at ages 30-34 for 758 (22.9%) offspring and 6,527 (22.5%) controls, at ages 35-39 for 236 (7.1%) offspring and 2,111 (7.3%) controls, after age 40 for 86 (2.6%) offspring and 757 (2.6%) controls, and missing for 101 (3.0%) offspring and 960 (3.3%) controls. Based on the ISCED 2011 international classification of education, educational level was categorised into seven groups of education ordered from lower to higher level: educational level missing, primary and lower secondary, upper secondary, post-secondary non-tertiary, short-cycle tertiary education, Bachelor’s or equivalent level, and Master’s/doctoral level or equivalent. (UNESCO, 2012).

*Offspring and grandchildren: Marriage, divorce and age at first child*

For individuals in Denmark alive in 1968 or later, marital status from 1968 up to July 2013, as well as start date and end date (if applicable) of status were available from the Danish Civil Registration System (Pedersen *et al*., 2006). For the analysis of marriages, the time scale for the marriage analysis was age, and the controls used were the same as in previous analyses, i.e. matched on birth year and sex and restricted to individuals born in Denmark. In the analyses of time to divorce, only individuals with known date of marriage were included, and the time scale was time since marriage. New controls were selected, matched on sex, birth year, and year of marriage, but otherwise randomly selected from the general Danish population, with one control per offspring and four controls per grandchild. The link between the offspring generation and their children, available from the Danish Civil Registration System, is considered complete for mothers born after 1935 and nearly complete for fathers born after 1945, conditional on their children being born after or surviving to 1973, and it enables calculation of age at first child.

**Results**

*Matched controls*

Of the 5,379 identified offspring, two were excluded from the analyses because they emigrated/died before April 2, 1968 when the Civil Registration System was implemented. In the matching of offspring with controls, which was made before any restriction to individuals born in Denmark, the 5,377 offspring were successfully matched 1:10 with 53,770 controls on sex and birth year. For the 9,938 grandchildren, where the 1:10 matching was on sex, birth year and on the grandchild’s LEF parents’s sex and birth year, the matching on the parents’ birth year was relaxed when a perfect match was not possible: of the 99,380 selected controls, 93,388 (93.4%) were matched perfectly, a further 5,180 (5.2%) matched on parental birth years with +/- 2 years, 586 (0.6%) controls had parental birth year 3-5 years from the grandchild’s parent’s birth year, 162 (0.2%) differed with 6-10 years and finally 64 (0.1%) controls differed more than 10 years.

In the comparison of divorce rates, a perfect 1:1 match of the 4,620 offspring on sex, birth year and year of marriage was obtained for 4,603 (99.6%) offspring, while the remaining 17 (0.4%) offspring were perfectly matched on sex and age at marriage, but with a birth year within 1-3 years from the offspring birth year. For the divorce rates of the 5,256 married grandchildren (1:4 matching), 20,026 (99.5%) of the 21,024 controls were perfectly matched on sex, birth year and year of marriage, while the remaining 98 controls were matched exactly on sex and age at marriage, but with a different birth year with the vast majority (91 controls) differing by 1 year.

*Age distribution of outcomes and individuals at risk*

Both for disease incidence and mortality, the distribution of outcomes and individuals at risk are presented in Figures S4 and S5. The figures show that for offspring vs. controls, the average number of individuals at risk varied from above 10,000 at the extremities of the age range to over 40,000 individuals in the central part of the age range. For grandchildren vs. controls, the corresponding average number of individuals at risk varied from over 50,000 at the lowest ages, increasing to at least 85,000 at the central ages, and, subsequently, decreasing to a number still well over 12,000 individuals. As for the number of outcomes at different ages, Figures S4 and S5 show that for all-cause mortality and higher incidence diseases, comparisons of offspring vs. controls and grandchildren vs. controls were fairly well-powered at most ages. However, for low incidence diseases, as well as for cause-specific diseases, the number of outcomes were more modest in some of the age intervals.

*Offspring: Disease incidence*

Since disease incidence was studied in the 35-year-period 1977-2011 and at ages 20-69, and 60 offspring and 781 controls died, emigrated or disappeared before 1977 or age 20 years, the study population for disease incidence in the offspring generation consisted of 5,273 offspring and 44,697 controls. The average and mean age at entry was 30 years for all 22 disease categories, and the time at risk was on average 31-32 years (median 34-35 years) for 18 of the categories, while, for the remaining four, it was above an average of 28 years (median above 33 years).

In the sub-strata of males and females, the HRs were very similar to the combined group, almost all of which were below 1 (exception: females, neoplasms, benign, with HR=1.01) and, for both sexes, most HRs were significantly lower (unadjusted: 15 of the 17 significant differences from the combined; Bonferroni-adjusted: for males 12 and for females 13 of the 14 significant differences from the combined analysis).

*Offspring: Cause-specific mortality*

In the study of cause-specific mortality, the study window for the offspring generation was ages 20-70 years in the 41-year- period 1970-2010, reducing the offspring sample by 17 to 5,316 offspring, and the controls by 221 resulting in 45,257 controls. Average age at entry was 25 years (median: 24 for offspring, 23 for controls years) and average time at risk was 36 years (median: 39 for offspring, 38 for controls years).

*Grandchildren: Disease incidence*

Since 12 controls emigrated and 70 controls and 7 grandchildren died before 1977, the study population for disease incidence consisted of 9,931 grandchildren and 99,298 controls. For all 22 diseases, the average age at entry was 5 years (median: 2 years) and for the majority of diseases the average time at risk was 30 years, and always above 26 years (median: mostly 34 years; always above 31 years).

The sex-specific HRs were similar to the combined group: for both males and females, 20 HRs (point estimates) were below 1 and two were close to 1, and for males, 10 HRs were significantly below 1 (Bonferroni correction, 22 tests: HR still significantly below 1 for 3 disease categories), while, for females, 12 HRs were significantly below 1 (Bonferroni correction, 22 tests: HR still significantly below 1 for 5 disease categories).

*Grandchildren: Cause-specific mortality*

For the grandchild generation, the restriction of the study of cause-specific mortality to the 38 years, period 1973-2010, did not exclude any individuals so that the entire available sample of 9,938 grandchildren and 99,380 controls were included in the analysis. Average age at entry was 2.7 years (median: 0 for both grandchildren and controls) and average time at risk was 31 years (median: 35 years for both grandchildren and controls).

*Offspring and grandchildren: disease incidence at ages 20-49*

In Figure S3, the comparison of disease incidence of offspring and grandchildren to their respective control groups are shown in the same graph using the same age window: 20-49 years. The figure shows that both offspring and grandchildren share a lower disease incidence compared to the general Danish population, whereas for most disease categories, the risk reduction is about half the size in the grandchildren compared to the offspring.

*Intergenerational similarity in disease occurrence*

In the sex-specific strata the results were similar: point estimates of HRs were all well below 1, and except for cardiovascular diseases among females, there was no significant difference between families with different familial histories of disease in the disease incidence advantage over the control sample for the general Danish population. Compared to their same-sexed controls, the health advantage was relatively larger among male LEF offspring compared to female LEF offspring: all HRs except two were lower among males, with the two exceptions very similar for males and females.

*Marriage and divorce incidence*

Of the 5,333 offspring born in Denmark, 68 (1.3%) had missing date of marriage compared to 1,001 (2.2%) among the 45,478 controls. The average time at risk among the offspring was 30.3 years (median 25.6 years) resulting in 4,620 marriages (87.7%) among the 5,265 offspring and among the remaining 44,477 controls the average time at risk was 29.6 years (median 24.8 years) with 39,106 marriages (87.9%). For the male offspring generation, the survival curves in Figure S8 (upper panel) show that for ages 18-29 years the proportion of married offspring was slightly lower than in the general population controls and slightly higher after age 30 years. The age-specific HRs show that up to age 23, offspring married less often than controls, between age 24 and 33 offspring married more frequently, and from age 34 onwards the marriage rates were similar with a slight tendency of higher marriage incidence among the offspring. For the female offspring generation (Figure S8, lower panel), the proportion of “never married” was slightly larger among the offspring. Given the overall slightly lower tendency to marry (relative to their controls) among females compared to male offspring, the age-specific marriage incidence rates among females had a pattern similar to the males with much lower marriage incidence among offspring at the earliest ages 16-19 years, and similar rates between female offspring and controls after age 22 years with perhaps a slight tendency of relatively lower marriage rates among offspring after age 36 years. Specifically, teenage marriages of offspring vs. controls resulted in HR=0.45 (95%-CI: 0.31-0.66) among males and HR=0.60 (95%-CI: 0.53-0.68) among females.

Of the 4,620 married offspring there were three (0.1%) with missing date of divorce, and this was also the case for four (0.1%) of the 4,620 matched controls. The average follow-up time for the 4,617 offspring was 31.7 years (median: 34.2 years) with 1,099 (23.8%) divorces during the study period, whereas for the 4,616 controls the average time at risk was 28.3 years (median: 29.6 years) and with 1470 (31.8%) divorces.

Divorce rates among male offspring were markedly lower than among their controls from 0 to 19 years after marriage (range of HRs: 0.50-0.86), but similar between the two groups from 20 years after marriage (Figure S9, upper panel). The divorce survival curves show a correspondingly much lower proportion of divorcees among offspring compared to the birth- and marriage year-matched male controls. The results for the female offspring generation (Figure S9, lower panel) were very similar: markedly lower divorce rates among female offspring up to 17 years after marriage (range of HRs: 0.44-0.95), a short spell at age 18-19 years with similar divorce rates among female offspring and controls followed by a return to lower divorce rates among offspring from age 20 years and onwards. Correspondingly, the divorce survival functions show a markedly higher proportion of non-divorcees among the female offspring at any time after marriage. Comparing the divorce rates within five years after marriage of offspring vs. controls resulted in HR=0.57 (95%-CI: 0.45-0.71) among males and HR=0.53 (95%-CI: 0.41-0.68) among females.

While all grandchildren had known dates of first marriage, a very small fraction of the 99,380 controls had missing dates of first marriage and were excluded (0.00%). For grandchildren, the average follow-up time was 29.9 years (median: 29.2) with 5,256 first marriages (52.9%), and for controls the average follow-up was 30.1 years (median: 29.1) resulting in 50,491 first marriages (50.8%). For marriages in the grandchild generation, the overall marriage rates both among males and female grandchildren were similar to, but slightly higher than, their respective controls (Figure S10, upper and lower panels). These overall rates reflected the age-specific rates where, for both males and females: the marriage rates among grandchildren at the lowest ages (below age 22) were lower than among their controls; at age 22-25 years they were similar among grandchildren and controls, and from age 26 years and onwards they were higher among male and female grandchildren, with a slight, relative decrease towards the null among male grandchildren after age 40 years. The marriage survival curves showed a similar or slightly lower proportion of married individuals among male and female grandchildren up to their mid/late twenties at which age the married proportion among the grandchildren increased slightly, but steadily faster than among the controls. Teenage marriages of grandchildren vs. controls resulted in HR=0.53 (95%-CI: 0.24-1.20) among males and HR=0.66 (95%-CI: 0.48-0.90) among females.

In the analysis of divorce in the grandchild generation, the 5,256 married grandchildren were matched (on sex, birth year and marriage year) to 21,024 married controls, one of which was excluded because marriage occurred after end of study. The average time at risk was 11.0 years (median: 9.8 years) for grandchildren with 1,110 divorces (21.1%) versus on average 10.6 years (median: 9.1 years) for controls with 5,159 divorces (24.5%). The divorce rates among males and female grandchildren were markedly lower in the first 10-15 years after marriage (range of HRs: males, 0-11 years: 0.68-0.94; females, 0-15 years: 0.59-0.92) after which the rates among the grandchildren were similar to (males after 12 years of marriage) or slightly higher (females after 18 years of marriage) than among the controls (Figure S11, upper and lower panels). The divorce survival curves show that the proportions of “not divorced” during the study period were moderately higher among the grandchildren, with the difference being largest around 12 years after marriage for males and around 16 years after marriage for females. After these ages, the difference between the survival curves decreased, but only very slightly. The divorce rates within five years after marriage of grandchildren vs. controls resulted in HR=0.74 (95%-CI: 0.62-0.88) among males and HR=0.78 (95%-CI: 0.67-0.90) among females.

*Number of children born to offspring and controls*

For all 5,333 offspring and 45,478 controls (born in Denmark), the number of children alive in 1973 or born after 1973 and before the end of 2010 were determined by the parent-child link from the Danish Civil Registration System. In Figure S12, the proportions of all male (upper panel) and female (lower panel) offspring and controls having zero, one, two, three, four, five, or six or more children are presented. Among male offspring, the proportions with zero and one child were lower than among the controls with correspondingly larger proportions among offspring having two and three children. The proportions having four, five, and six or more children were similar among offspring and controls. For females, the differences between offspring and controls were smaller with a lower proportion among offspring with one child and a slightly lower proportion with two children but with a correspondingly larger proportion with three children. The proportions with zero, four, five, and six or more children were similar among female offspring and their controls.

*Age at first child*

Of the 5,333 offspring born in Denmark, there were 838 (17.7%) offspring without children resulting in a total of 4,495 parent offspring. Of the 45,478 controls, the non-parents amounted to 7,604 (18.9%) and a remaining 37,874 parent controls. Figure S13 shows the distribution function of age at first child for offspring of Danish longevity-enriched families and their controls conditional upon being parents within the study period (upper panel: males; lower panel: females). The frequencies of offspring parents at the youngest ages were lower than those of the controls: for male offspring: up to age 25 years, and for female offspring: up to age 22 years. For both male and female parent offspring, the accumulated difference amounted to about 12% fewer parents who had their child at the aforementioned ages, respectively, than was the case for the controls, and, specifically, among parents the proportion of teenage parentage was 2.2% among male offspring compared to 4.3% among male controls (HR=0.50; 95%-CI: 0.38-0.67), whereas for female offspring this number was 7.4% compared to 16.5% among female controls (HR=0.42; 95%-CI: 0.36-0.49). For male offspring, the median age at first child was 27.4 years (95%-CI: 27.1-27.5 years) compared to 26.2 years (95%-CI: 26.1-26.3 years), and similarly, for female offspring, the median age at first child was 24.7 years (95%-CI: 24.5-24.9 years) compared to 23.6 years (95%-CI: 23.5-23.7 years).

**References**

American Cancer Society. Cancer Facts & Figures 2019. Atlanta: American Cancer Society; 2019

Jensen VM, Rasmussen AW. The Danish Education Registers. *Scand J Public Health* 2011; **39** (7 Suppl): 91–94.

Lynge E, Sandegaard JL, Rebolj M. The Danish national patient register. *Scand J Public Health*. 2011;39(7 Suppl):30–33.

Pedersen CB. The Danish Civil Registration System. *Scand J Public Health* 2011; **39**(7 Suppl): 22-5.

Pedersen CB, Gøtzsche H, Møller JO, Mortensen PB. The Danish Civil Registration System. A cohort of eight million

persons. *Dan Med Bull* 2006; **53**: 441–49.

Schmidt M, Schmidt SAJ, Sandegaard JL, Ehrenstein V, Pedersen L, Sørensen HT. The Danish National Patient Registry: a review of content, data quality, and research potential. *Clinical Epidemiology* 2015; **7**: 449-490.

SNBH (2017a) The Swedish National Board of Health Vol. 2017.

SNBH (2017b) The Swedish National Board of Health: Conversion tables for ICD Vol. 2017.

UNESCO (2012) International Standard Classification of Education ISCED 2011. Montreal, Quebec, Canada: UNESCO Institute for Statistics

| **Supplement Table S1A. Characteristics of LEF-siblings.** | | | | |
| --- | --- | --- | --- | --- |
|  |  | LEF-siblings | | |
|  |  |  |  |  |
|  |  |  |  |  |
| No. families | | 659 | | |
|  | With ≥ two nonagenarian siblings | > 99% of the families | | |
|  | Average sibship size (per LEF-family)^†^ | | 6.03 | |
|  | Average percentage of nonagenarians | 57.7% | |  |
|  |  |  |  |  |
|  |  | Siblings identified in population register | | |
|  |  |  | |  |
| Number of individuals^†,††^ | | 3,972 | |  |
|  |  |  |  |  |
| Birth year | |  |  |  |
|  | Average | 1912.8 | |  |
|  | Q1-Q3 |  | 1909-1917 | |
|  | p5-p95 |  | 1902-1925 | |
|  |  |  |  |  |
| Females | | 2,162 | | 54.4% |
|  |  |  |  |  |
| Number of offspring^†††^ | | 5,388 | |  |
|  | Average offspring sibship size |  | 1.36 |  |
|  |  |  |  |  |
|  |  | **Number** | | **Percent** |
| Alive at end of follow-up (July 1, 2013) | | 484 | | 12.2% |
|  | Above age 90 |  | 346 | 71.5% |
|  | Average age on July 1, 2013 |  | 93.7 |  |
|  |  |  |  |  |
| Deaceased by end of follow-up | | 3,479 | | 87.6% |
|  | Above age 90 |  | 1,711 | 49.2% |
|  | Average age at death |  | 87.2 |  |
|  |  |  |  |  |
| Emigrated/disappeared/inactive | | 9 | | 0.2% |
|  | Average age at end date |  | 66.1 |  |
|  |  |  |  |  |
|  |  |  |  |  |
| ^†^3,972 is the number of siblings identified in the Danish Civil Registration System, which entails being alive and living in Denmark on or after April 2, 1968. The sibship size of 7.2 in the discussion section in the main manuscript refers to all siblings including those dying or emigrating before April 2, 1968. The siblings are born in the beginning of the 20^th^ century and at that time 10-15% of all newborns died before age 5 according to Statistics Denmark. | | | | |
| ^††^On April 2, 1968, the average age among siblings is about 55 years. | | | | |
| ^†††^The 5,388 offspring here is the number arrived at when counting the number of offspring of each LEF-siblings. However, it corresponds to 5,379 unique individuals some of whom have both parents from LEF-families but from distinct families. Moreover, two of these offspring were not followed in Danish registers. | | | | |

| **Supplement Table S1B. Characteristics of LEF-offspring, LEF-grandchildren and their respective controls.** | | | | | | | | | | | | | | | | | |  | |  |
| --- | --- | --- | --- | --- | --- | --- | --- | --- | --- | --- | --- | --- | --- | --- | --- | --- | --- | --- | --- | --- |
|  |  | Offspring generation | | | | | | |  |  | Grandchild generation | | | | | | | |  | |
| Birth year | | LEF-offspring | | |  | Controls | | |  |  | LEF-grandchildren | | |  | Controls | | | |  | |
|  | Average | 1946.9 | | |  | 1946.5 | | |  |  | 1975.6 | | |  | 1975.6 | | | |  | |
|  | Q1-Q3 | 1941-1953 | | |  | 1941-1952 | | |  |  | 1968-1982 | | |  | 1968-1982 | | | |  | |
|  | p5-p95 | 1934-1962 | | |  | 1933-1962 | | |  |  | 1960-1995 | | |  | 1960-1995 | | | |  | |
|  |  | **Number** | |  |  | **Number** | |  |  |  | **Number** | |  |  | **Number** | |  | |  | |
| LEF families | | 634 | |  |  |  |  |  |  |  | 627 | |  |  |  |  |  | |  | |
| Study population | | 5,333 | |  |  | 45,478 | |  |  |  | 9,938^†^ | |  |  | 99,380 | |  | |  | |
|  | Females |  | 2,606 |  |  |  | 22,967 |  |  |  |  | 4,851 |  |  |  | 48,510 |  | |  | |
|  | Grandchildren |  | 10,363 |  |  |  | 84,691 |  |  |  |  |  |  |  |  |  |  | |  | |
|  | (incl. born outside Denmark & multiple births) | | **Average** |  |  |  | **Average** |  |  |  |  |  |  |  |  |  |  | |  | |
|  | Grandchildren per offspring |  | 1.94 |  |  |  | 1.86 |  |  |  |  |  |  |  |  |  |  | |  | |
|  | Offspring’s age at birth |  | 29.2 | **Median** |  |  | 28.0 |  |  |  |  |  |  |  |  |  |  | |  | |
|  |  |  | **Average** |  |  |  |  |  |  |  |  |  |  |  |  |  |  | |  | |
|  | Age of LEF-parent (i.e. sibling) at birth | | 33.1 | 32.7 |  |  |  |  |  |  |  |  |  |  |  |  |  | |  | |
|  |  |  |  |  |  |  |  |  |  |  |  |  |  |  |  |  |  | |  | |
| Marriage analysis (n; % missing) | | 5,265 | | 1.3% |  | 44,477 | | 2.2% |  |  | 9,938 | | 0.0% |  | 99,377 | | 0.0% | |  | |
|  | Marriages (n; % married individuals) |  | 4,628 | 87.9% |  |  | 39,171 | 88.1% |  |  |  | 5,256 | 52.9% |  |  | 50,491 | 50.8% | |  | |
|  |  |  |  |  |  |  |  |  |  |  |  |  |  |  |  |  |  | |  | |
| Matched divorce analysis (n; % missing) | | 4,616 | | 0.3% |  | 4,617 | | 0.0% |  |  | 5,256 | | 0.0% |  | 20,995 | | 0.0% | |  | |
|  | Divorces (n; % divorced of married indiv.) |  | 1,099 | 23.81% |  |  | 1,470 | 31.84% |  |  |  | 1,110 | 21.1% |  |  | 5,125 | 24.4% | |  | |
|  |  |  |  |  |  |  |  |  |  |  |  |  |  |  |  |  |  | |  | |
| Mortality analysis (n; % of study pop.) | | 5,316 | | 99.7% |  | 45,257 | | 99.5% |  |  | 9,938 | | 100.0% |  | 99,380 | | 100.0% | |  | |
|  | Deaths (n; % of mortality sample) |  | 380 | 7.1% |  |  | 6,528 | 14.4% |  |  |  | 138 | 1.4% |  |  | 1,962 | 2.0% | |  | |
|  |  |  |  |  |  |  |  |  |  |  |  |  |  |  |  |  |  | |  | |
| Disease analysis (n; % of study pop.) | | 5,271 | | 98.8% |  | 44,697 | | 98.3% |  |  | 9,931 | | 99.9% |  | 99,298 | | 99.9% | |  | |
|  | |  |  |  |  |  |  |  |  |  |  |  |  |  |  |  |  | |  | |
|  |  | **Percent** | | **95%-CI** | | **Percent** | | **95%-CI** | |  | **Percent** | | **95%-CI** | | **Percent** | | **95%-CI** | | | |
| Survival probability for study period | | 87.8% | | 86.4-89.0% | | 77.2% | | 76.7-77.8% | |  | 96.6% | | 95.7-97.4% | | 95.4% | | 95.1-95.7% | | | |

^†^Excluding multiple births and children born outside Denmark.

| **Supplement Table S2. Categories of diseases and causes of death with ICD-10 and ICD-8 codes - based on ICD-10 categories of disease and with corresponding ICD-8 codes.** | | | | | | | | | |
| --- | --- | --- | --- | --- | --- | --- | --- | --- | --- |
|  |  |  |  |  |  |  | Cause of death categories | | |
| Order |  | Short name of ICD-10 category | ICD-10 codes |  | ICD-8 codes |  | Offspring |  | Grandchildren |
|  |  |  |  |  |  |  |  |  |  |
| 1 |  | Bacterial infections | A000 - A999 |  | 0 - 469, 600 - 689, 800 - 1049 |  | *Rare cause*^†^ |  | *Rare cause*^†^ |
| 2 |  | Non-bacterial infections | B000 - B999 |  | 500 - 579, 700 - 799, 1100 - 1369 |  | *Rare cause* |  | *Rare cause* |
| 3 |  | Neoplasms, malignant^††,†††^ | C000 - C979, D000-D099 |  | 1400 – 2099 |  | Neoplasms, malignant |  | Neoplasms, malignant |
| 4 |  | Neoplasms, benign | D100 - D369 |  | 2100 – 2289 |  | *Rare cause* |  | *Rare cause* |
| 5 |  | Neoplasms, unknown / uncertain | D370 - D489 |  | 2300 - 2399 |  | *Rare cause* |  | *Rare cause* |
| 6 |  | Anaemia | D500 - D899 |  | 2800 - 2890 |  | *Rare cause* |  | *Rare cause* |
| 7 |  | Endocrine diseases | E000 - E900 |  | 2400 - 2799 |  | Endocrine |  | *Rare cause* |
| 8 |  | Mental and behavioural disorders | F000 - F999 |  | 2900-3099, 3100 - 3150 |  | Mental disorders |  | *Rare cause* |
| 9 |  | Neurological diseases | G000 - G989 |  | 3200 - 3580 |  | Neurological |  | Neurological |
| 10 |  | Eye diseases | H000 - H599 |  | 3600 - 3790 |  | *Rare cause* |  | *Rare cause* |
| 11 |  | Ear diseases | H600 - H959 |  | 3800 - 3890 |  | *Rare cause* |  | *Rare cause* |
| 12 |  | Cardiovascular diseases | I000 - I999 |  | 4000 - 4580 |  | Cardiovascular |  | Cardiovascular |
| 13 |  | Respiratory diseases | J000 - J998 |  | 4600 - 5190 |  | Respiratory |  | *Rare cause* |
| 14 |  | Gastro-intestinal diseases | K000 - K930 |  | 5200 - 5770 |  | Gastro-intestinal |  | *Rare cause* |
| 15 |  | Dermatological disorders | L000 - L998 |  | 6800 - 7090 |  | *Rare cause* |  | *Rare cause* |
| 16 |  | Rheumatic diseases | M000 - M999 |  | 7100 - 7380 |  | *Rare cause* |  | *Rare cause* |
| 17 |  | Kidney and urinary diseases | N000 - N999 |  | 5800 - 6290 |  | *Rare cause* |  | *Rare cause* |
| 18 |  | Neonatal diseases | P000 - P969 |  | 7600 - 7790 |  | *Rare cause* |  | Neonatal |
| 19 |  | Congenital malformations | Q000 - Q999 |  | 7400 - 7590 |  | *Rare cause* |  | Congen. malf. |
| 20 |  | Symptoms not elsewhere classified | R000 - R999 |  | None |  | *Other* |  | *Other* |
| 21 |  | Injury | S000 - T983 |  | None (N800 - N999) |  | *Rare cause* |  | *Rare cause* |
| 22 |  | External causes | V000 - Y989 |  | 8000 - 9999 (E800 - E999) |  | External causes |  | External causes |
| 23 |  |  | Any other code |  | Any other code |  | *Other* |  | *Other* |
| 24 |  |  | NA |  | NA |  | *Missing* |  | *Missing* |

^†^A cause of death which is rare (in the observed age range) in the study population, where *rare* corresponds to less than 5 events among either LEFs or controls.

^††^In tables for cancer only, and when studying associations with family history of cancer, non-melanoma skin cancer has been excluded from the (malignant) cancers which comprises ICD-8 codes: 204-209\(173, 208), and ICD-10 codes: CXX.X \ (C44, C46.0), D09.0-1, D30.1-9, D32-33, D35.2-4, D41.1, D42-43, D44.3-5, D45-46, D47.0-1, D47.3-9

^†††^Tobacco-related cancer is the subcategory of malignant cancer comprising cancer of lip, oral cavity, salivary glands, pharynx, oesophagus, stomach, colon, rectum and anus, liver, pancreas, larynx, lung, cervix uteri, kidney, bladder and other/unspecified urinary organs, and acute leukaemia (ICD-8: 140-151, 153-154, 155.0-1, 157, 161, 162.1-2, 180, 188-189, 204.0, 205.0, 206.0, 207.0, 207.2; ICD-10: C00-16, C18-22, C25, C32-34, C53, C64-68, C91.0, C92.0, C93.0, C94.0, C94.2, C94.4-5, D09.0-1, D30.1-9, D41.1-9)

| **Supplement Table S3. Categories of the diseases and causes of death with ICD-10, ICD-9 and ICD-8 codes - based on ICD-10 categories of disease and with subsequent conversions firstly from ICD-10 to ICD-9 and, subsequently, from ICD-9 to ICD-8 codes using conversion tables from the Swedish National Board of Health and Welfare.** | | | | | | |
| --- | --- | --- | --- | --- | --- | --- |
|  |  |  |  |  |  |  |
| Order^†^ |  | ICD-10 |  | ICD-9 |  | ICD-8 |
|  |  |  |  |  |  |  |
| 1 |  | A000 - A999 |  | 0000 - 0499 \ {0340,0394,0402},0541, 0547,0600-0699, 071,073,0748,0760-0770,0779,0781-0783, 0786-0788,0799, 0800-0839,0870-0992, 0994-1049, 1310-1319,1364-1365,1447,2901,2941, 3230A- 3232A,3315A-3316A, 4828, 5281,6390, 670,7713,7855,7907 |  | 0000 - 0499 \ {0122,0190-0199,0340,044},0500, 054, 0600-0689, 071, 073,0749-076,0780,0789,0791-0793, 0798,0800-0839,0880-1049, 1118-1119,113,1174-1179, 131,136,2901,292-,2932,2934,2939,2941, 2948-2949, "309-",3331-3339,3479, 429,460,473,481,4829,5281,597, 6313, "644-"-"645-","661-", 670, 711,7149,7239,7572, 7829,Y34- |
| 2 |  | B000 - B999 |  | 0119-0120,0160,0170,0172,0176,0394-0399, 0498,0500-0540,0542-0579, 0625,0700-0709, 0720-0729,0740-075, 0770-0781,0784-0788, 0798-0799, 0840-0865, 1100-130,1320-1349, 1362-1363,1368-1398,2795-2796,3221, 3314, 3501,410-414,415,416,417,418,4809, 5791,790, 7908,791, 792,793, 798, 799, 9990 |  | 011,0121,0158,016-0171,0179,0190-0199,044,0500-0579, 0629,065-066,070,072,0740-075,077-0791,0794-0799, 0840-0879,0999,110-1300, 1302-1309,132-1349,136, 2690,3209, 323,3479,351,"410-"-"414-",426, 429,460, 470,480,4829,484, 711,7149,7572,9990,9992-9993 |
| 3 |  | C000 - C979, D000-D099 |  | 1400-2089,2300-2349,2362,2386-2387, 2389, 2732-2733,2898,7573, UNDEF |  | 1400-1991,2000-209,2113,2115,220,2268,227-2319,2322-2376,2380-2399,2560,2589,2754-2759,279,2872,2899, "382-",5287,5297,569,600, 6071,6211,6959,702,709,7571-7572, 7579,7598,"978-"-"979-" |
| 4 |  | D100 - D369 |  | 2100-2299,2381 |  | 1419,1709,1719,2000,2029,2101-2109,2110-"219-",220-228, 2305-2306, 2320-2321,2370,2387,2399,"241-",2421, 246,251-2520,2530,2532,2552,2560,2570, 2580,5262,600, 610 |

^†^Order refers to the order of the first 22 disease categories in Table S1.

| **Supplement Table S3 *(continued)*. Categories of the diseases and causes of death with ICD-10 codes, ICD-9, and ICD-8 codes** | | | | | | |
| --- | --- | --- | --- | --- | --- | --- |
| Order |  | ICD-10 |  | ICD-9 |  | ICD-8 |
|  |  |  |  |  |  |  |
| 5 |  | D370 - D489 |  | 2299,2350-2376,2379-2385,2387-2399, 2580-2588,2849-2850,2888,2898 |  | 1719,181-1820,1830,1890,191,1929,1991,2029,2079-209, 2102,2113, 2116,2123,213,215-217,"219-"-2210,2219-2220, 2230-2231,2233, 2259-2260,2262-2399,250-251, 2550,2552, 2560,2570,2581,2759,284-2850,2872,2899, 600,7571-7572 |
| 6 |  | D500 - D899 |  | 135,2727,2730,2732,2776-2778,2790-2793,2798-2890,2894-2899,5294, 7091,7440A |  | 135,2079-208,209,254,2690,2708,2720-2729,2738,2750-2751, 2754-2759,279-2871,2873-2890,2894-2899,3819, 4461,5290, 5294,5719,573, 7080,709,7570,9976 |
| 7 |  | E000 - E900 |  | 2400-2579,2581-2681,2689-2729,2738-2739, 2750-2788,2799,2814, 2882, 2898,2941,3300-3301,5698,5939,6268,6281A,6868,7090,7598, 7834, 7836, 7906,9976,9999 |  | 2029,209,2420-2531,2539-2651,2659-268,2699-274,2752-2753,2759-2810,2814,2871,2890,2899,"292-",2932,2934, 2939, 2941, 2948-2949,3321,3330-3331,3479,569,581, 5932,5935, 6159,6259,6266, 6269-628,6869,7080,709, 7149,739,7570, 7588-7589,7592,7598-7599, 7789,7829, 7880,7885-7887,7889, 7938,7960,828,9611-9612,9620, 9623,9636,9909,9974,9989, 9995,9999-Y00 |
| 8 |  | F000 - F999 |  | 2900-319,7800,7836,7846, UNDEF, V400-V409, V417, V652 |  | 066,2789,2900-3019,3021-"315-",3459,3479,"382-",6779, 7800-7801,7803-7804,7815-7816,7832,7889,7902,7960-7961, 8509, 9650,9670,9679,969-971,9745,9778-"979-", 9899 |
| 9 |  | G000 - G989 |  | 0498,1369,2901,2941,303,3074,3078,3200-3220, 3222-3229,3233A-326, 3302A-3314,3317A-3599, 3623, 3664A,3686A, 3886,4338,4349-4370, 4378-4379,4478, 4580,479,7249,7282, 7360,7428,7805, 7813,7840,7860, 9899, 9970 |  | 0360,065,136,2734,2738,2750,2901,"292-",2932,2934, 2939,2941,2948-2949,3008,3031-3039,3051-3053,3055-3056,3059, 3062,3064,3068-3069,308,"309-",3200-3329, 3331-3339,340-358,373,3770,3789, 3819,3879,3929,  "432-"-"433-","435-", 4369-4389,442,4469-447,4580, 4829,573,596,7149-715,7172-7179,7239,726-7270,7287, 7289-729, 7330-7339,738,7438-7439,7789,7803-7804, 7806,7814,7816-7818,7832, 7871,791, 794,7960,9673-9679,9769,9779,984,9851,9899,9909,9970, 9976,9989, 9991,9995,9999 |
|  | | | | | | |
| **Supplement Table S3 *(continued)*. Categories of the diseases and causes of death with ICD-10 codes, ICD-9, and ICD-8 codes** | | | | | | |
| Order |  | ICD-10 |  | ICD-9 |  | ICD-8 |
|  |  |  |  |  |  |  |
| 10 |  | H000 - H599 |  | 2641,2722,2751,3008,3600-3799,979,V410-V411 |  | 0903,"216-",2601-2608,2720,2733,279,3001,3008-3009, 3479, 360-"379-",4569-457,6929,6954,7011,704-705, 7444,7448-7449,7810-7812,7818, 8700-"871-",930,9620, 9859,9989 |
| 11 |  | H600 - H959 |  | 3800-3899,6800,9151,V412-V413 |  | 380-3819,3830-"389-",6800,6929,7278,7805,7811,7813, 920,"951-",9613, 9989 |
| 12 |  | I000 - I999 |  | 0998,2891-2893,2899,3418,3526,390-413,4140-4149,4150-4151,4160-4169, 4170-4179,4200A-4349,436-4449, 4470-4589,4591-4599,6958,9971- 9972, 9979 |  | 0999,2891-2893,2899,344,3453,3479,3499-350,356-357, 390-398,"410-"-"434-",4360-"442-",4430-4431,4438-4441, 4444-4450,4469-4589, 6959,7059,709, 7467,7473,7824, 9611,9681, 9971,9973,9975-9976, 9989,9999 |
| 13 |  | J000 - J998 |  | 0114,0340,460-4789,4800-5199,5308, 6800,6820, 7281,7309,7332,7339, 7380,7388,7991,9973 |  | 010-011,0122,0340,114,1173,2109,213,355,460-5199, 5309, 537,"661-",6800,6820,7202-7203,7230-7239,729, 7339,738, 7484,7824,7832-7833,7871,7960,9909,9981, 9985-9986,9989, 9999 |
| 14 |  | K000 - K930 |  | 0402,4590,5200-5280,5282-5361,5368-5799, 6820,6868,9974, V416, V473 |  | 0399,054,2029,211,2113,2690-2699,3032,"413-",4403, 4442, 447,4589, 5200-5280,5282-5779,6820-6821,6869, 707,7803, 7814,7842,7844-7845, 7855,7857,7873,7960, 9651,9779,9906, 9979,9987-9989,9999 |
| 15 |  | L000 - L998 |  | 0390,1100,1360,2507,6800-7062, 7068-7099, 7821,7854,9491,9851, UNDEF |  | 110,1118-1119,113,136,2029,"216-",250,2708,"382-", 4459, 6800-704, 7050-707,7089-709,7572,7882,9611-9612,9735, 9779-"979-",9809,9851, 9899-9900,9909,9995 |
| 16 |  | M000 - M999 |  | 0090,0993,1361,2520,2682,2740-2749,2754, 2794,2941,3379,3596A, 3599, 410,4460-4467, 4476,4478, 7100-7196,7198-7399,7561,7565, 9588,9849,9967,9979,9995, UNDEF |  | ----,0089-0090,0092-0099,136,213,215,2520,2652, 2734, 2738, 274, 2759,279,"292-",2932,2934,2939,2941,2948-2949,334, 352-353,355, 357-358,"382-","410-"-"411-", 426,429,4460-447, 508,5159,592,6869,710-732, 7331-739, 7561,7564-7566,771, 7803,7871-7874,7885,7889,"829-" ,844,8729,"978-"-"979-", 984, 9959,9974-9977,9989,9995 |

| **Supplement Table S3 *(continued)*. Categories of the diseases and causes of death with ICD-10 codes, ICD-9, and ICD-8 codes** | | | | | | |
| --- | --- | --- | --- | --- | --- | --- |
| Order |  | ICD-10 |  | ICD-9 |  | ICD-8 |
|  |  |  |  |  |  |  |
| 17 |  | N000 - N999 |  | 0994,3446,4462,5800-6280,6282-6299, 7533, 7880, 7910,7919,8796,9840-9841, 9849-9850, 9853,9855-9858, 9975,9979, 9999,V474-V475 |  | 0999,136,203,"219-"-2212,2219,2232,2238,2520,2738, 3499, 357,443,4461,4589,580-6070,6072-6210,6212-6299, 678,7289, 7526,7528, 7533,7598,7860,7862,7866-7867, 7886,7890-7891, 7893,7899,792,7960,8630, 8679,8790, 8797,9612-9613,984-9850,9853-9859,9906-9909,9974, 9989, 9995,9999 |
| 18 |  | P000 - P969 |  | 3588,5119,6268,7470,7479,7533,7600-7712,7714-7735,7741-7799 |  | 0039,011,0122,0270,0389,0399,043,054,056,070,0780,0795, 0849,0909,112,1301,136,208,2422,250-251,2521,2589, 2699, 280,2871,288,2894-2899,3302,3489,360,368,470, 486,5112, 5601,567,5719,599,6110,6259,6266,6269,6821, 6861-6869, 6950,6959,7089-709,7330-7331,7442,7470, 7479,7524,7533, 7572,7598,7600-7709,7710-7799,7802, 7841,7883, 7885-7886, 7889,7901,9602,9990,9998 |
| 19 |  | Q000 - Q999 |  | 2377,2871,3526,5268,5289,5306,5511, 5521,5531,6208,6238,7283,7400-7599 |  | 2119-2120,"216-",220-2211,224,227-228,2320,2738,2873, 334, 344, 3479,350,356-357,370,3779,3789,425,5240, 5242,5262, 5269, 5280, 5287,5289,5309,5511,5513,5531, 569,6159,6169, 6259,6292,6295,6299,6959,729,7339,738, 740-7466,7468-7523, 7525-7599,7789,7824 |

| **Supplement Table S3 *(continued)*. Categories of the diseases and causes of death with ICD-10 codes, ICD-9, and ICD-8 codes** | | | | | | |
| --- | --- | --- | --- | --- | --- | --- |
| Order |  | ICD-10 |  | ICD-9 |  | ICD-8 |
|  |  |  |  |  |  |  |
| 20 |  | R000 - R999 |  | 0341,2599,2639,2738,2750,2754,2766,2872,2888,2989,3009,3079,3128-3129,3151,319,3229,3319,3681,4278, 4590,4598,460,5110,5277,5362-5368, 5640,5688,5698,5718,5742,5938,5997-5998,6869-690,6940,6958,7038, 7063,7090,7093,7098,7197, 7298-7299,7543,7800-7804,7806-7879, 7881-7899,7900-7907,7909,7910-7919,7920-7929, 7930-797,7980-7989, 7990-7999,9970,V401, V414-V416 |  | 0341,0389,203,2079,209,2232,2422,250,2581,2589,268,2699, 2708,2732,2734,2738,274,2752-2753,2759,277,2789-280, 2839,2870,2890,2899, 2982,299,3009,3055-3056, 3060-3065, 3069,308,"315-",3209,323,3459, 3479,351, 370,3779,3789, 4249,4279,429,443,4459,4580-460,508, 5110,5192-5199,5277,5279,5299,535-537,5640,569, 5719, 573-574,576,5932,5935-594,596,599,6079,6293,6869-690, 693,6959,7011,703-704,7059,7063,709,7149,7179-718, 729,7339,738,7556,7789,7800-7805,7807-7810,7812, 7814-7816,7818-7841,7843-7844,7846-7856,7858-7865, 7870-7872,7874-7876,7881-7886,7888-7890, 7892-792, 7938,794-7960, 7962-7969,9610-9613,9620,9681, 9770, 9779,9900,9909,9943,9970, 9989,9991,9995,9999,Y00 |
| 21 |  | S000 - T983 |  | 0059,2650,2941,3803,4430,5212,6291,6931,7098,7175,8000-9972,9976, 9980-9999, UNDEF |  | 0059,261,"292-",2932,2934,2939,2941,2948-2949,3209, 323,3479,3499,3789,"382-",3879,"431-",4430,4432,447, 4589, 5212,6296,6299,6869, 6923,6925,6929,7080,709, 7241,738, 7528,7889,7969-"827-","828-"-999 |
| 22 |  | V000 - Y989 |  | E8000-E999, UNDEF, e8263 |  | 4432,6928,E800-807,810-823,825-827,830-838,840-845, 8500-35,8539-43,8549-56,8559-64,8568-75,8578-86,8589-877,880-887,890-94,896-910,9100,9109,911-912,9130, 9139,914-919, 9200-9202,9208-9209,"921-",9210-9211, 9218,"922-",9229-9232,9238-39,924,9250-9251,9258-9261, 927-929,9300-9302,9309-9313,9318-9323,9328-9336,9338-9347,9349,935-936,940-946, 950-978, 980-999 |

| **Supplement Table S4. Offspring of Danish LEF (n=5,273) versus control sample (n=44,697) matched on sex and birth year: comparison of incidence of disease-specific hospitalization for 22 main categories of disease between age 20 and 69 years, 1977-2011^†^.** | | | | | | | | | | | | | | | | | | | | | | | | | | | | | | | | |  |
| --- | --- | --- | --- | --- | --- | --- | --- | --- | --- | --- | --- | --- | --- | --- | --- | --- | --- | --- | --- | --- | --- | --- | --- | --- | --- | --- | --- | --- | --- | --- | --- | --- | --- |
|  | LEF offspring vs. controls - restricted to ages 20-69 years | | | | | | | | | | | | | | | | | | | | | | | | | | | | | | | |  |
|  | Males and females | | | | |  | LEFs vs. controls | | | | | | | | | | | | | | | | | | | | | | | | | |  |
|  | LEFs | |  | Controls | |  | Males and females | |  | Males^††^ | | | | | | | | |  | | Females^††^ | | | | | | | | | | | |  |
|  | Disease events | |  | Disease events | |  | HR | 95%-CI |  | HR |  | 95%-CI | | | | | | |  | | HR | | | |  | | 95%-CI | | | | | |  |
| Bacterial infections | 187 |  |  | 2,165 |  |  | **0.70** | 0.60-0.80 |  | **0.69** |  | 0.56-0.85 | | | | | | |  | | **0.70** | | | |  | | 0.57-0.87 | | | | | |  |
| Non-bacterial infections | 58 |  |  | 677 |  |  | **0.70** | 0.54-0.91 |  | **0.69** |  | 0.48-0.99 | | | | | | |  | | 0.71 | | | |  | | 0.47-1.08 | | | | | |  |
| Neoplasms, malignant | 505 |  |  | 5,708 |  |  | **0.72** | 0.65-0.80 |  | **0.63** |  | 0.54-0.73 | | | | | | |  | | **0.80** | | | |  | | 0.70-0.90 | | | | | |  |
| Neoplasms, benign | 492 |  |  | 4,258 |  |  | 0.98 | 0.90-1.08 |  | 0.86 |  | 0.70-1.06 | | | | | | |  | | 1.02 | | | |  | | 0.92-1.13 | | | | | |  |
| Neoplasms, unkn./uncert.^†††^ | 110 |  |  | 1,215 |  |  | **0.77** | 0.64-0.94 |  | 0.77 |  | 0.46-1.29 | | | | | | |  | | **0.77** | | | |  | | 0.63-0.95 | | | | | |  |
| Anaemia | 72 |  |  | 822 |  |  | **0.71** | 0.55-0.90 |  | **0.62** |  | 0.44-0.90 | | | | | | |  | | 0.78 | | | |  | | 0.58-1.06 | | | | | |  |
| Endocrine diseases | 233 |  |  | 2,849 |  |  | **0.67** | 0.59-0.77 |  | **0.58** |  | 0.46-0.72 | | | | | | |  | | **0.75** | | | |  | | 0.63-0.88 | | | | | |  |
| Mental disorders | 133 |  |  | 2,082 |  |  | **0.52** | 0.43-0.62 |  | **0.53** |  | 0.42-0.67 | | | | | | |  | | **0.50** | | | |  | | 0.38-0.67 | | | | | |  |
| Neurological diseases | 242 |  |  | 2,998 |  |  | **0.66** | 0.58-0.75 |  | **0.59** |  | 0.49-0.72 | | | | | | |  | | **0.72** | | | |  | | 0.61-0.87 | | | | | |  |
| Eye diseases | 98 |  |  | 976 |  |  | 0.83 | 0.68-1.01 |  | 0.87 |  | 0.65-1.15 | | | | | | |  | | 0.78 | | | |  | | 0.58-1.06 | | | | | |  |
| Ear diseases | 90 |  |  | 837 |  |  | 0.89 | 0.73-1.09 |  | 0.97 |  | 0.73-1.29 | | | | | | |  | | 0.82 | | | |  | | 0.61-1.10 | | | | | |  |
| Cardiovascular diseases | 936 |  |  | 10,494 |  |  | **0.71** | 0.66-0.76 |  | **0.66** |  | 0.60-0.73 | | | | | | |  | | **0.78** | | | |  | | 0.70-0.86 | | | | | |  |
| Respiratory diseases | 511 |  |  | 5,398 |  |  | **0.77** | 0.70-0.84 |  | **0.76** |  | 0.67-0.86 | | | | | | |  | | **0.77** | | | |  | | 0.67-0.89 | | | | | |  |
| Gastro-intestinal diseases | 1,011 |  |  | 10,132 |  |  | **0.80** | 0.75-0.86 |  | **0.83** |  | 0.76-0.90 | | | | | | |  | | **0.77** | | | |  | | 0.70-0.86 | | | | | |  |
| Dermatological disorders | 228 |  |  | 2,229 |  |  | **0.83** | 0.72-0.96 |  | **0.82** |  | 0.68-0.99 | | | | | | |  | | 0.85 | | | |  | | 0.68-1.06 | | | | | |  |
| Rheumatic diseases | 829 |  |  | 8,021 |  |  | **0.84** | 0.78-0.91 |  | **0.85** |  | 0.77-0.95 | | | | | | |  | | **0.83** | | | |  | | 0.75-0.92 | | | | | |  |
| Kidney and urinary diseases | 1,102 |  |  | 10,150 |  |  | **0.90** | 0.84-0.96 |  | 1.00 |  | 0.88-1.13 | | | | | | |  | | **0.86** | | | |  | | 0.80-0.94 | | | | | |  |
| Neonatal diseases^††††^ | * |  |  | 25 |  |  | * | * |  | * |  | * | | | | | | |  | | * | | | |  | | * | | | | | |  |
| Congenital malformations | 70 |  |  | 648 |  |  | 0.90 | 0.70-1.15 |  | 0.81 |  | 0.56-1.17 | | | | | | |  | | 0.97 | | | |  | | 0.70-1.35 | | | | | |  |
| Symptoms, nec^†††††^ | 541 |  |  | 5,451 |  |  | **0.79** | 0.72-0.86 |  | **0.76** |  | 0.67-0.86 | | | | | | |  | | **0.82** | | | |  | | 0.73-0.93 | | | | | |  |
| Injury | 732 |  |  | 6,724 |  |  | **0.87** | 0.80-0.94 |  | **0.89** |  | 0.81-0.99 | | | | | | |  | | **0.83** | | | |  | | 0.74-0.94 | | | | | |  |
| External causes | 683 |  |  | 7,023 |  |  | **0.80** | 0.74-0.87 |  | **0.82** |  | 0.74-0.90 | | | | | | |  | | **0.77** | | | |  | | 0.68-0.88 | | | | | |  |
| ^†^Risk time range was 148,478.3-168,045.1 person-years for offspring and 1,202,756.0-1,392,824.1 person-years for controls | | | | | | | | | | | | | | | | | | | | | | | | | | | | | | | | | |
| ^††^ For males: 2,693 LEF offspring vs. 22,137 controls; for females: 2,580 LEF offspring vs. 22,560 controls | | | | | | | | | | | | | | | | | | | | | | | | | | | | | | | | | |
| ^†††^Neoplasms of unknown or uncertain type | | | | | | | | | | | | | | | | | | | | | | | |  | |  | |  |  |  |  |  | |
| ^††††^Numbers not shown because less than 5 events | | | | | | | | | | | | |  |  |  |  |  |  | |  | |  |  |  | |  | |  |  |  |  |  | |
| ^†††††^Not elsewhere classified | | | | | | | | | | | | |  |  |  |  |  |  | |  | |  |  |  | |  | |  |  |  |  |  | |

| **Supplement Table S5. Offspring of Danish LEF (n=5,273) versus control sample (n=44,697) matched on sex and birth year: comparison of cause-specific mortality for 10 main categories of cause of death between age 20 and 69 years, 1970-2010^†^.** | | | | | | | | | | | | | | | | | | | | | | | | | | | |
| --- | --- | --- | --- | --- | --- | --- | --- | --- | --- | --- | --- | --- | --- | --- | --- | --- | --- | --- | --- | --- | --- | --- | --- | --- | --- | --- | --- |
|  |  | LEF offspring vs. controls - restricted to ages 20-69 years | | | | | | | | | | | | | | | | | | | | | | | | | |
|  |  |  |  |  |  | | |  |  | |  | | |  | |  | | | |  | | |  | | | | |
|  |  | Males and females | | | | | | | | | | | | | | | | | | | | | | | | | |
|  |  |  |  | | |  |  |  | | | |  |  | |  | |  | | | |  | | |  | | | |
|  |  | LEFs | | | |  | Controls | | | | |  | LEFs vs. controls | | | | | | | | | | | | | | |
|  |  |  |  | | |  |  |  | | | |  |  | | | | |  |  | | |  | | |  | | |
|  |  |  |  | | |  |  |  | | | |  |  | | | | |  |  | | |  | | |  | | |
|  |  | Cause specific deaths | | | |  | Cause specific deaths | | | | |  | HR | | | | |  | 95%-CI | | | | | | | | |
| Neoplasms, malignant | | 162 | |  | |  | 2,492 | | |  | |  | **0.54** | | | | |  | 0.45 | | | | | | | - | 0.63 |
| Endocrine diseases | | 15 | |  | |  | 187 | | |  | |  | 0.65 | | | | |  | 0.38 | | | | | | | - | 1.13 |
| Mental disorders | | 14 | |  | |  | 206 | | |  | |  | **0.54** | | | | |  | 0.32 | | | | | | | - | 0.92 |
| Neurological diseases | | 9 | |  | |  | 155 | | |  | |  | **0.48** | | | | |  | 0.23 | | | | | | | - | 1.00 |
| Cardiovascular diseases | | 62 | |  | |  | 1,255 | | |  | |  | **0.40** | | | | |  | 0.30 | | | | | | | - | 0.52 |
| Respiratory diseases | | 19 | |  | |  | 337 | | |  | |  | **0.46** | | | | |  | 0.28 | | | | | | | - | 0.73 |
| Gastro-intestinal diseases | | 16 | |  | |  | 401 | | |  | |  | **0.32** | | | | |  | 0.20 | | | | | | | - | 0.53 |
| External causes | | 53 | |  | |  | 799 | | |  | |  | **0.55** | | | | |  | 0.41 | | | | | | | - | 0.73 |
| Rare or missing causes | | 15 | |  | |  | 325 | | |  | |  | **0.38** | | | | |  | 0.22 | | | | | | | - | 0.65 |
| Other causes | | 15 | |  | |  | 371 | | |  | |  | **0.33** | | | | |  | 0.20 | | | | | | | - | 0.54 |
| Any cause | | 380 | |  | |  | 6,528 | | |  | |  | **0.48** | | | | |  | 0.42 | | | | | | | - | 0.53 |

^†^Risk time for the 5,316 offspring accumulated to 193,380 person-years, and for the 45,257 controls amounted to 1,607,736 person-years.

| **Supplement Table S6. Grandchildren of Danish LEF (n=9,931) versus control sample (n=99,298) matched on sex, birth year and one parent's birth year: comparison of incidence of disease-specific hospitalization for 22 main categories of disease between age 0 and 49 years, 1977-2011^†^.** | | | | | | | | | | | | | | | | | |
| --- | --- | --- | --- | --- | --- | --- | --- | --- | --- | --- | --- | --- | --- | --- | --- | --- | --- |
|  | LEF grandchildren vs. controls - restricted to ages 0-49 years | | | | | | | | | | | | | | | | |
|  | Males and females | | | | |  | LEFs vs. controls | | | | | | | | | | |
|  | LEFs | |  | Controls | |  | Males and females | | |  | Males^††^ | | |  | Females^††^ | | |
|  | Disease events | |  | Disease events | |  | HR |  | 95%-CI |  | HR |  | 95%-CI |  | HR |  | 95%-CI |
| Bacterial infections | 455 |  |  | 5,762 |  |  | **0.78** |  | 0.71-0.86 |  | **0.81** |  | 0.71-0.94 |  | **0.75** |  | 0.65-0.87 |
| Non-bacterial infections | 275 |  |  | 3,069 |  |  | 0.89 |  | 0.79-1.01 |  | 0.90 |  | 0.77-1.06 |  | 0.88 |  | 0.73-1.06 |
| Neoplasms, malignant | 140 |  |  | 1,608 |  |  | 0.87 |  | 0.72-1.03 |  | 0.82 |  | 0.62-1.08 |  | 0.90 |  | 0.72-1.13 |
| Neoplasms, benign | 297 |  |  | 2,984 |  |  | 0.99 |  | 0.88-1.12 |  | 0.96 |  | 0.77-1.20 |  | 1.01 |  | 0.87-1.16 |
| Neoplasms, unkn./uncert.^†††^ | 44 |  |  | 534 |  |  | 0.82 |  | 0.60-1.12 |  | 0.91 |  | 0.42-1.95 |  | 0.80 |  | 0.57-1.14 |
| Anaemia | 93 |  |  | 830 |  |  | 1.12 |  | 0.90-1.38 |  | 1.08 |  | 0.80-1.46 |  | 1.15 |  | 0.87-1.54 |
| Endocrine diseases | 251 |  |  | 2,702 |  |  | 0.92 |  | 0.81-1.05 |  | 0.93 |  | 0.76-1.14 |  | 0.92 |  | 0.77-1.10 |
| Mental disorders | 271 |  |  | 3,508 |  |  | **0.76** |  | 0.67-0.88 |  | **0.77** |  | 0.65-0.92 |  | **0.75** |  | 0.62-0.91 |
| Neurological diseases | 314 |  |  | 3,854 |  |  | **0.81** |  | 0.72-0.91 |  | **0.77** |  | 0.65-0.91 |  | **0.84** |  | 0.72-0.99 |
| Eye diseases | 149 |  |  | 1,609 |  |  | 0.92 |  | 0.77-1.10 |  | 1.05 |  | 0.83-1.35 |  | 0.80 |  | 0.61-1.04 |
| Ear diseases | 279 |  |  | 2,769 |  |  | 1.00 |  | 0.89-1.14 |  | 0.98 |  | 0.83-1.15 |  | 1.04 |  | 0.87-1.25 |
| Cardiovascular diseases | 345 |  |  | 3,885 |  |  | **0.88** |  | 0.79-0.98 |  | 0.92 |  | 0.79-1.07 |  | **0.84** |  | 0.72-0.98 |
| Respiratory diseases | 1,716 |  |  | 18,053 |  |  | **0.94** |  | 0.89-0.99 |  | 0.99 |  | 0.93-1.06 |  | **0.89** |  | 0.82-0.96 |
| Gastro-intestinal diseases | 1,421 |  |  | 15,999 |  |  | **0.88** |  | 0.82-0.93 |  | **0.89** |  | 0.82-0.96 |  | **0.86** |  | 0.79-0.94 |
| Dermatological disorders | 455 |  |  | 4,675 |  |  | 0.97 |  | 0.88-1.07 |  | 0.97 |  | 0.85-1.10 |  | 0.97 |  | 0.85-1.11 |
| Rheumatic diseases | 718 |  |  | 8,067 |  |  | **0.88** |  | 0.81-0.96 |  | **0.89** |  | 0.79-0.99 |  | **0.88** |  | 0.78-0.98 |
| Kidney and urinary diseases | 1,077 |  |  | 11,735 |  |  | **0.90** |  | 0.85-0.96 |  | 0.90 |  | 0.80-1.00 |  | **0.90** |  | 0.84-0.98 |
| Neonatal diseases | 277 |  |  | 3,327 |  |  | **0.83** |  | 0.72-0.95 |  | **0.82** |  | 0.69-0.98 |  | 0.83 |  | 0.69-1.01 |
| Congenital malformations | 459 |  |  | 4,671 |  |  | 0.98 |  | 0.89-1.08 |  | 1.05 |  | 0.94-1.18 |  | 0.87 |  | 0.73-1.03 |
| Symptoms, nec^††††^ | 876 |  |  | 10,408 |  |  | **0.83** |  | 0.77-0.89 |  | **0.78** |  | 0.69-0.88 |  | **0.86** |  | 0.79-0.94 |
| Injury | 1,460 |  |  | 15,809 |  |  | **0.91** |  | 0.86-0.97 |  | **0.93** |  | 0.86-1.00 |  | **0.89** |  | 0.82-0.98 |
| External causes | 1,517 |  |  | 16,688 |  |  | **0.90** |  | 0.85-0.95 |  | **0.92** |  | 0.85-0.98 |  | **0.87** |  | 0.80-0.95 |

| ^†^Risk time range was 263,014.6-301,322.9 person-years for grandchildren and 2,582,545.7-2,999,773.1 person-years for controls | | | | | | | | | | | |
| --- | --- | --- | --- | --- | --- | --- | --- | --- | --- | --- | --- |
| ^††^ For males: 5,084 LEF grandchildren vs. 50,826 controls; for females: 4,847 LEF grandchildren vs. 48,472 controls | | | | | | | | | | | |
| ^†††^Neoplasms of unknown or uncertain type | | | | | | | | | |  |  |
| ^††††^Not elsewhere classified |  |  |  |  |  |  |  |  |  |  |  |

| **Supplement Table S7. Grandchildren of Danish LEF versus control sample (10:1) matched on sex, birth year and one parent's birth year: comparison of cause-specific mortality for 9 main categories of cause of death between age 0 and 49 years, 1973-2010^†^.** | | | | | | | | | | | |
| --- | --- | --- | --- | --- | --- | --- | --- | --- | --- | --- | --- |
|  | LEF grandchildren vs. controls - restricted to ages 0-49 years | | | | | | | | | | |
|  |  |  |  |  |  |  |  |  |  |  |  |
|  | Males and females | | | | | | | | | | |
|  |  |  |  |  |  |  |  |  |  |  |  |
|  | LEFs | |  | Controls | |  | LEFs vs. controls | | | | |
|  |  |  |  |  |  |  |  |  |  |  |  |
|  | Cause specific deaths | |  | Cause specific deaths | |  | HR |  | lb |  | Ub |
|  |  |  |  |  |  |  |  |  |  |  |  |
| Neoplasms, malignant | 25 |  |  | 283 |  |  | 0.88 |  | 0.59 | - | 1.30 |
| Neurological diseases | 6 |  |  | 74 |  |  | 0.81 |  | 0.36 | - | 1.84 |
| Cardiovascular diseases | 8 |  |  | 153 |  |  | 0.52 |  | 0.26 | - | 1.04 |
| Neonatal diseases | 10 |  |  | 131 |  |  | 0.76 |  | 0.38 | - | 1.52 |
| Congenital malformations | 10 |  |  | 146 |  |  | 0.68 |  | 0.36 | - | 1.29 |
| External causes | 49 |  |  | 714 |  |  | **0.68** |  | 0.51 | - | 0.91 |
| Rare or missing causes | 19 |  |  | 315 |  |  | **0.60** |  | 0.37 | - | 0.98 |
| Other cause | 11 |  |  | 146 |  |  | 0.75 |  | 0.41 | - | 1.36 |
| Any causes | 138 |  |  | 1,962 |  |  | **0.70** |  | 0.59 | - | 0.84 |

^†^Risk time for the 9,938 grandchildren accumulated 313,397 person-years, and for the 99,380 controls amounted to 3,123,225 person-years.

| **Supplement Table S8. Distribution of LEF families in tertiles of disease occurrence in siblings, 1977-2011: subjects, disease events and rates^†^.** | | | | | | | | | | | | | | | | | | | | | | |
| --- | --- | --- | --- | --- | --- | --- | --- | --- | --- | --- | --- | --- | --- | --- | --- | --- | --- | --- | --- | --- | --- | --- |
|  | Family disease history |  | LEF siblings | | | | | | | | | |  | Offspring | | | |  | Controls | | | |
|  |  |  | Families | | Siblings | | Disease events | | Min rate | | Max rate | |  | Offspring | | Disease events | |  | Controls | | Disease events | |
| Cardio-vascular diseases | Lower tertile |  | 212 |  | 1,207 |  | 344 |  | 0 |  | 1,892 |  |  | 1,794 |  | 286 |  |  | 15,301 |  | 3,649 |  |
|  | Middle tertile |  | 211 |  | 1,285 |  | 662 |  | 1,895 |  | 3,135 |  |  | 1,877 |  | 329 |  |  | 15,834 |  | 3,573 |  |
|  | Upper tertile |  | 211 |  | 1,212 |  | 861 |  | 3,152 |  | 51,263 |  |  | 1,602 |  | 321 |  |  | 13,562 |  | 3,272 |  |
|  | Combined |  | 634 |  | 3,704 |  | 1,867 |  |  |  |  |  |  | 5,273 |  | 936 |  |  | 44,697 |  | 10,494 |  |
| Respira-tory diseases | Lower tertile |  | 212 |  | 1,176 |  | 67 |  | 0 |  | 686 |  |  | 1,769 |  | 143 |  |  | 14,989 |  | 1,757 |  |
|  | Middle tertile |  | 211 |  | 1,328 |  | 320 |  | 687 |  | 1,429 |  |  | 1,812 |  | 197 |  |  | 15,364 |  | 1,904 |  |
|  | Upper tertile |  | 211 |  | 1,200 |  | 571 |  | 1,431 |  | 6,223 |  |  | 1,692 |  | 171 |  |  | 14,344 |  | 1,737 |  |
|  | Combined |  | 634 |  | 3,704 |  | 958 |  |  |  |  |  |  | 5,273 |  | 511 |  |  | 44,697 |  | 5,398 |  |
| Cancer | Lower tertile |  | 212 |  | 1,090 |  | 43 |  | 0 |  | 560 |  |  | 1,721 |  | 164 |  |  | 14,724 |  | 1,927 |  |
|  | Middle tertile |  | 211 |  | 1,296 |  | 280 |  | 563 |  | 1,182 |  |  | 1,845 |  | 171 |  |  | 15,411 |  | 1,856 |  |
|  | Upper tertile |  | 211 |  | 1,318 |  | 531 |  | 1,182 |  | 4,613 |  |  | 1,707 |  | 169 |  |  | 14,562 |  | 1,865 |  |
|  | Combined |  | 634 |  | 3,704 |  | 854 |  |  |  |  |  |  | 5,273 |  | 504 |  |  | 44,697 |  | 5,648 |  |
| Tobacco-related cancer^††^ | Lower tertile |  | 318 |  | 1,578 |  | 0 |  | 0 |  | 0 |  |  | 2,350 |  | 87 |  |  | 20,064 |  | 1,182 |  |
|  | Middle tertile |  | 158 |  | 1,182 |  | 171 |  | 239 |  | 891 |  |  | 1,768 |  | 69 |  |  | 14,749 |  | 801 |  |
|  | Upper tertile |  | 158 |  | 944 |  | 299 |  | 893 |  | 6,879 |  |  | 1,155 |  | 34 |  |  | 9,884 |  | 589 |  |
|  | Combined |  | 634 |  | 3,704 |  | 470 |  |  |  |  |  |  | 5,273 |  | 190 |  |  | 44,697 |  | 2,576 |  |
| Mental and behaviour-al disorders^††^ | Lower tertile |  | 311 |  | 1,632 |  | 0 |  | 0 |  | 0 |  |  | 2,379 |  | 136 |  |  | 20,262 |  | 2,026 |  |
|  | Middle tertile |  | 161 |  | 1,169 |  | 180 |  | 230 |  | 925 |  |  | 1,651 |  | 112 |  |  | 13,865 |  | 1,515 |  |
|  | Upper tertile |  | 162 |  | 903 |  | 316 |  | 926 |  | 4,049 |  |  | 1,243 |  | 104 |  |  | 10,570 |  | 1,069 |  |
|  | Combined |  | 634 |  | 3,704 |  | 496 |  |  |  |  |  |  | 5,273 |  | 352 |  |  | 44,697 |  | 4,610 |  |

^†^Rates are presented per 100,000 person-years.

^††^Since, for each of tobacco-related cancer and mental and behavioural disorders, half of the families had no siblings with the disease/disorder in question, the lower tertile comprised half of the families, the middle and upper “tertiles” comprised the 3^rd^ and 4^th^ quartile.

| **Supplement Table S9. Intergenerational similarity in disease occurrence from LEF siblings to LEF offspring for five disease categories.** | | | | | | | | | | | | | | | | |
| --- | --- | --- | --- | --- | --- | --- | --- | --- | --- | --- | --- | --- | --- | --- | --- | --- |
|  |  |  | LEF offspring vs. controls - by family disease history | | | | | | | | | | | | | |
|  |  |  |  |  |  |  |  |  |  |  |  |  |  |  |  |  |
|  |  |  | Males and females | | | |  | Males | | | |  | Females | | | |
|  | Family disease history† |  |  |  |  |  |  |  |  |  |  |  |  |  |  |  |
|  |  |  |  |  |  |  |  |  |  |  |  |  |  |  |  |  |
|  |  |  | HR | Lower |  | Upper |  | HR | Lower |  | Upper |  | HR | Lower |  | Upper |
|  |  |  |  |  |  |  |  |  |  |  |  |  |  |  |  |  |
| Cardiovascular diseases | Lower tertile |  | 0.62 | 0.54 |  | 0.70 |  | 0.61 | 0.51 |  | 0.72 |  | 0.63 | 0.52 |  | 0.76 |
|  | Middle tertile |  | 0.74 | 0.66 |  | 0.83 |  | 0.66 | 0.57 |  | 0.77 |  | 0.84 | 0.70 |  | 1.00 |
|  | Upper tertile |  | 0.78 | 0.69 |  | 0.88 |  | 0.72 | 0.61 |  | 0.86 |  | 0.86 | 0.73 |  | 1.02 |
|  | Combined |  | 0.71 | 0.66 |  | 0.76 |  | 0.66 | 0.60 |  | 0.73 |  | 0.78 | 0.70 |  | 0.86 |
| Respiratory diseases | Lower tertile |  | 0.65 | 0.54 |  | 0.79 |  | 0.67 | 0.53 |  | 0.84 |  | 0.64 | 0.47 |  | 0.86 |
|  | Middle tertile |  | 0.83 | 0.72 |  | 0.96 |  | 0.81 | 0.65 |  | 0.99 |  | 0.87 | 0.70 |  | 1.06 |
|  | Upper tertile |  | 0.80 | 0.68 |  | 0.94 |  | 0.80 | 0.64 |  | 1.00 |  | 0.80 | 0.63 |  | 1.02 |
|  | Combined |  | 0.77 | 0.70 |  | 0.84 |  | 0.76 | 0.67 |  | 0.86 |  | 0.77 | 0.67 |  | 0.89 |
| Cancer | Lower tertile |  | 0.71 | 0.60 |  | 0.84 |  | 0.61 | 0.47 |  | 0.79 |  | 0.79 | 0.65 |  | 0.96 |
|  | Middle tertile |  | 0.74 | 0.63 |  | 0.88 |  | 0.66 | 0.52 |  | 0.84 |  | 0.81 | 0.65 |  | 1.00 |
|  | Upper tertile |  | 0.74 | 0.61 |  | 0.90 |  | 0.68 | 0.52 |  | 0.90 |  | 0.79 | 0.62 |  | 0.99 |
|  | Combined |  | 0.73 | 0.66 |  | 0.81 |  | 0.65 | 0.56 |  | 0.76 |  | 0.79 | 0.70 |  | 0.90 |
| Tobacco-related cancer^††^ | Lower tertile |  | 0.60 | 0.48 |  | 0.76 |  | 0.56 | 0.42 |  | 0.76 |  | 0.65 | 0.47 |  | 0.90 |
|  | Middle tertile |  | 0.69 | 0.53 |  | 0.89 |  | 0.71 | 0.50 |  | 1.00 |  | 0.66 | 0.46 |  | 0.96 |
|  | Upper tertile |  | 0.47 | 0.33 |  | 0.66 |  | 0.42 | 0.26 |  | 0.68 |  | 0.53 | 0.32 |  | 0.86 |
|  | Combined |  | 0.60 | 0.51 |  | 0.70 |  | 0.57 | 0.47 |  | 0.70 |  | 0.63 | 0.51 |  | 0.78 |
| Mental and behavioural disorders^††^ | Lower tertile |  | 0.54 | 0.45 |  | 0.65 |  | 0.52 | 0.41 |  | 0.67 |  | 0.56 | 0.43 |  | 0.74 |
|  | Middle tertile |  | 0.59 | 0.48 |  | 0.71 |  | 0.50 | 0.38 |  | 0.64 |  | 0.70 | 0.53 |  | 0.93 |
|  | Upper tertile |  | 0.78 | 0.63 |  | 0.98 |  | 0.69 | 0.52 |  | 0.92 |  | 0.90 | 0.67 |  | 1.20 |
|  | Combined |  | 0.61 | 0.54 |  | 0.68 |  | 0.55 | 0.47 |  | 0.64 |  | 0.69 | 0.59 |  | 0.81 |

^†^Categorized on tertiles of the crude disease rate in each LEF family based on the proband generation, so that e.g. the lower tertile corresponds to the families with the lowest occurrence of the disease in question among the proband generation.

^††^Since, for each of tobacco-related cancer and mental and behavioural disorders, half of the families had no siblings with the disease/disorder in question, the lower tertile comprised half of the families, the middle and upper “tertiles” comprised the 3^rd^ and 4^th^ quartiles (see Supplement Table S8 for exact numbers).

**Supplement Table S10. Wealth indicators in the 1916 Census for the G0 generation of 645 Longevity-Enriched Families (LEF) and 358 Control families.**

|  | **LEF** | **Control** |
| --- | --- | --- |
| **Income** (DKK)  Mean  SD  Median  Range  N | 1,793  1,915  1,300  0 – 17,435  494 | 2,061  5,377  1,198  0 – 80,500  266 |
| **Wealth** (DKK)  Mean  SD  Median  Range  N | 8,994  22,157  4000  0 – 407,200  449 | 9,266  16,790  3,500  0 – 185,272  240 |
| **State Tax** (DKK)  Mean  SD  Median  Range  N | 36.21  94.36  10.55  0 – 1,312  371 | 48.45  137.31  13.87  0 – 1,315  183 |
| **Municipal Tax** (DKK)  Mean  SD  Median  Range  N | 61.26  111.68  28.90  0 – 1,157  523 | 67.29  177.65  24.00  0 – 1,890  288 |

N is varying due to missing values. All differences are statistically non-significant (p>0.2 by t-test with unequal variance and Mann-Whitney test).





**Figure S1:** Study population flow chart for the analysis of disease incidence in the LEF proband generation, and the analyses of disease incidence and cause-specific mortality in the LEF offspring and LEF grandchild generation.


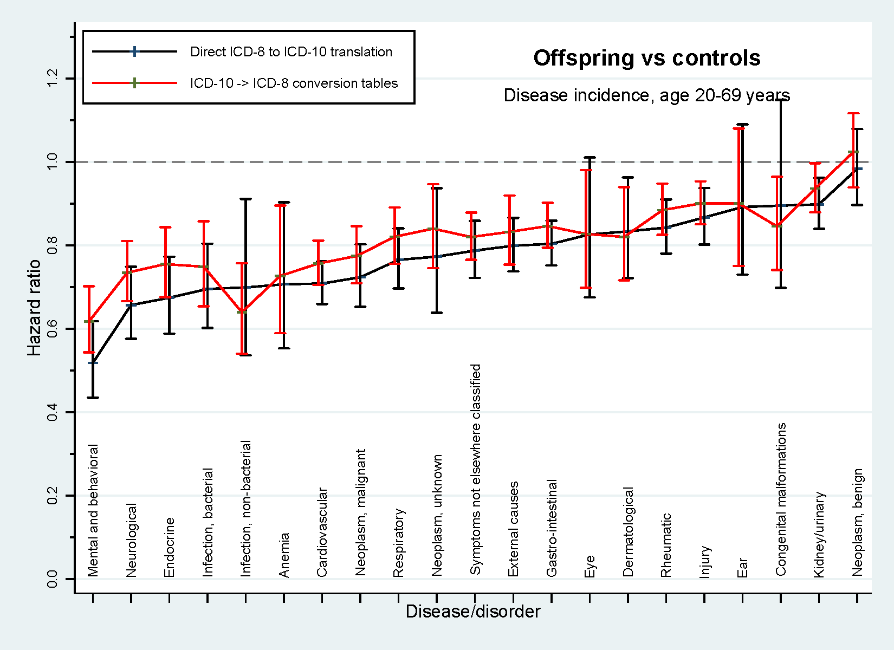

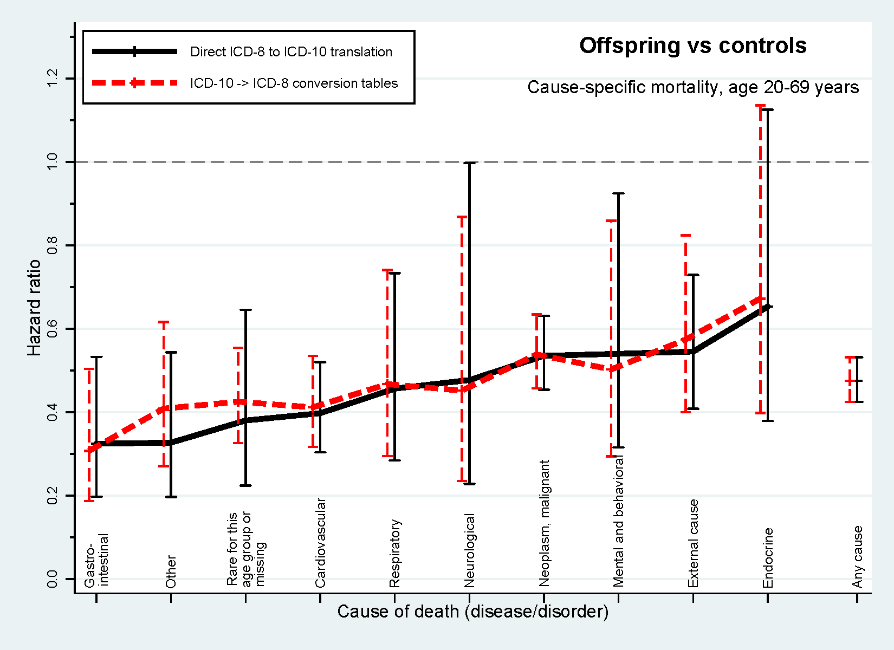

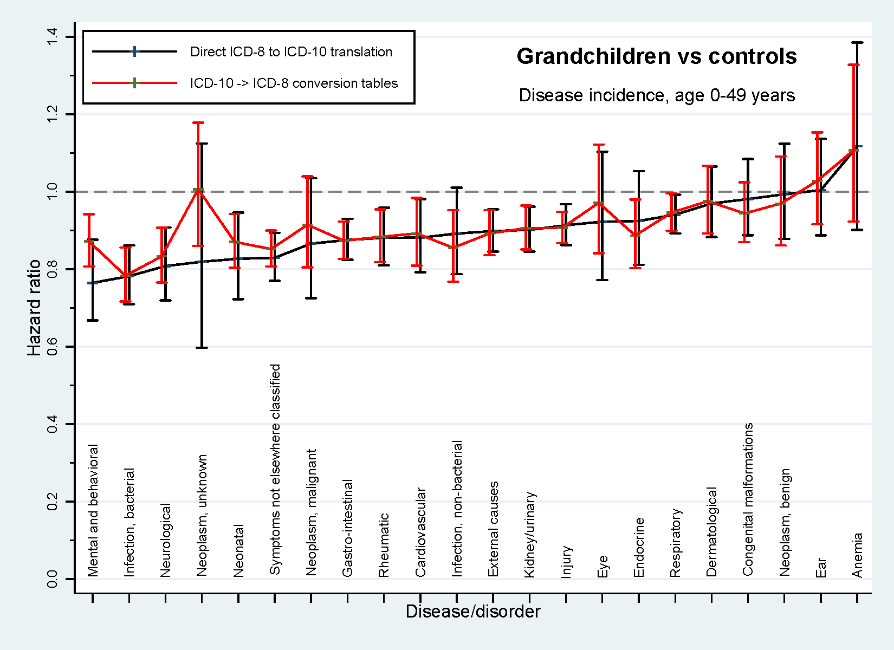

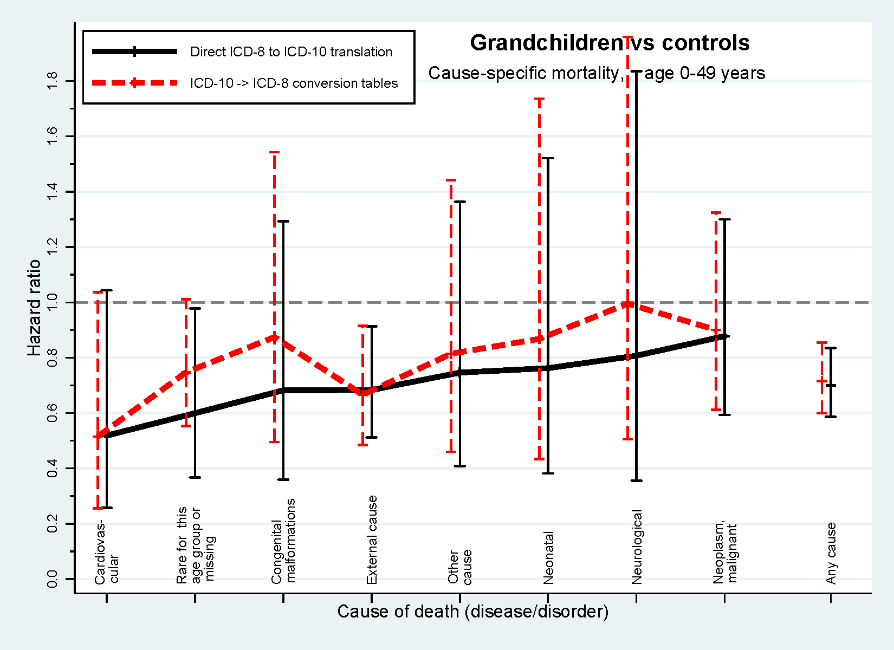


**Figure S2.** Offspring (and grandchildren) of Danish longevity-enriched families vs. age and sex (and parental age)-matched controls from a random sample of the general Danish population: comparison of disease incidence for 22 major disease categories and cause-specific mortality for ten (eight) cause-of-death categories. Disease and cause of death categories based on major ICD-10 categories and where the subsequent corresponding ICD-8 categories were based either on direct ICD-8 to ICD-10 translation (black lines) or via conversion tables from ICD-10 to ICD-9 and ICD-9 to ICD-8 available from the Swedish National Board of Health and Welfare (red lines).

**Note**: The sole purpose of lines connecting point estimates is to ease the comparison of the patterns of HRs based on the direct ICD-8 to ICD-10 translation to that based on the conversion tables, while the fact that the x-axis is categorical means that the connecting lines do not represent values of HR between the different categories the four panels of Figure S2.


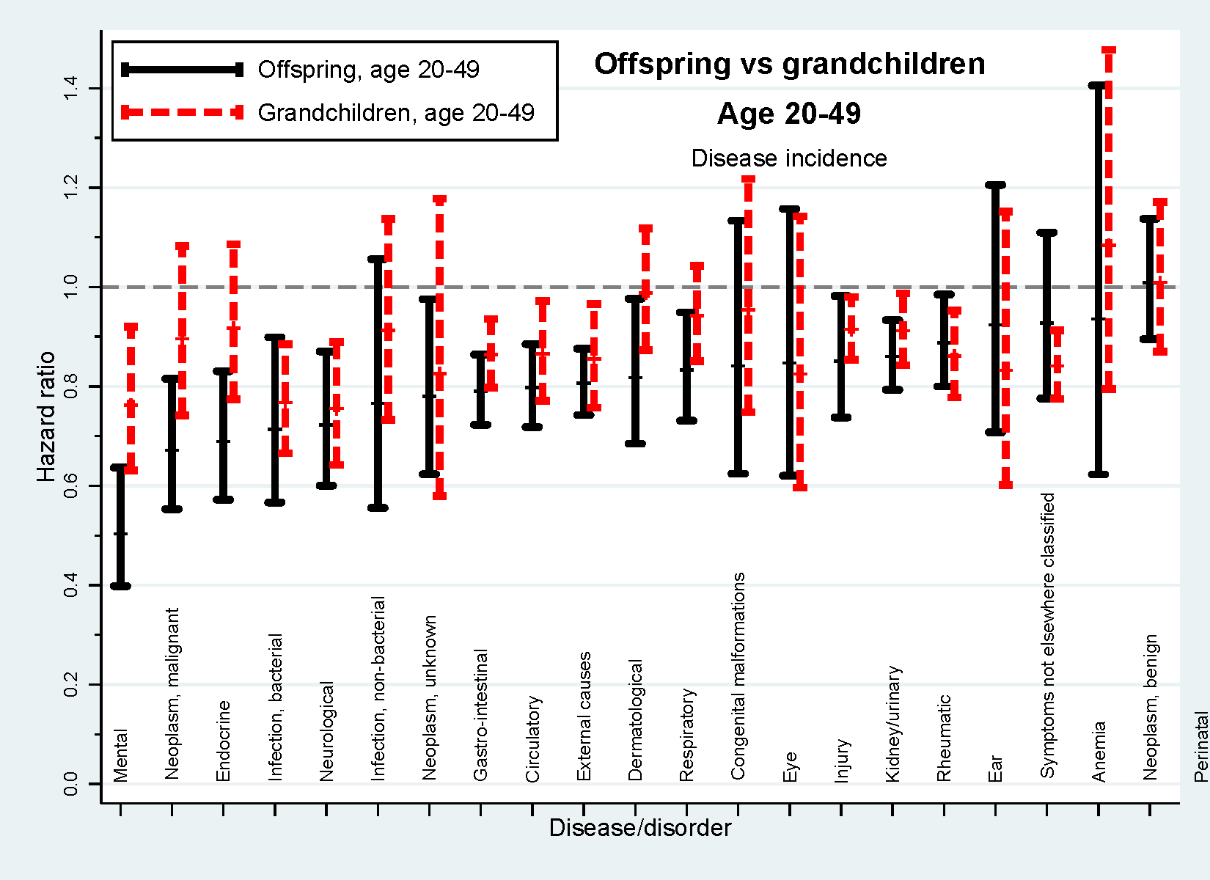


**Figure S3.** Offspring (and grandchildren) of Danish longevity-enriched families vs. age and sex (and parental age)-matched controls from a random sample of the general Danish population: comparison of disease incidence for 22 major disease categories. For both offspring and grandchildren, the age window of the comparison to the matched controls is restricted to ages 20-49 years to enable a better intergenerational comparison.


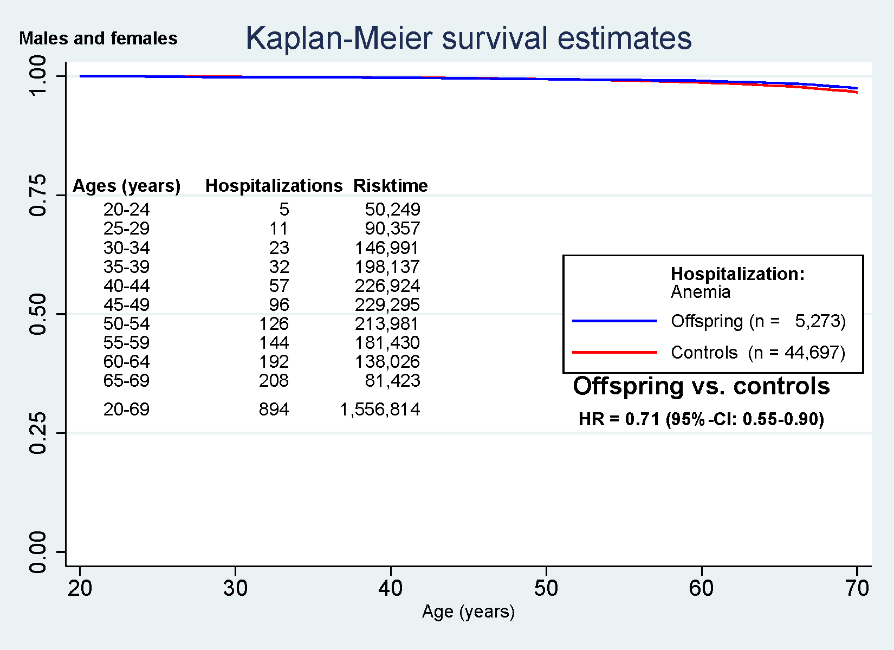

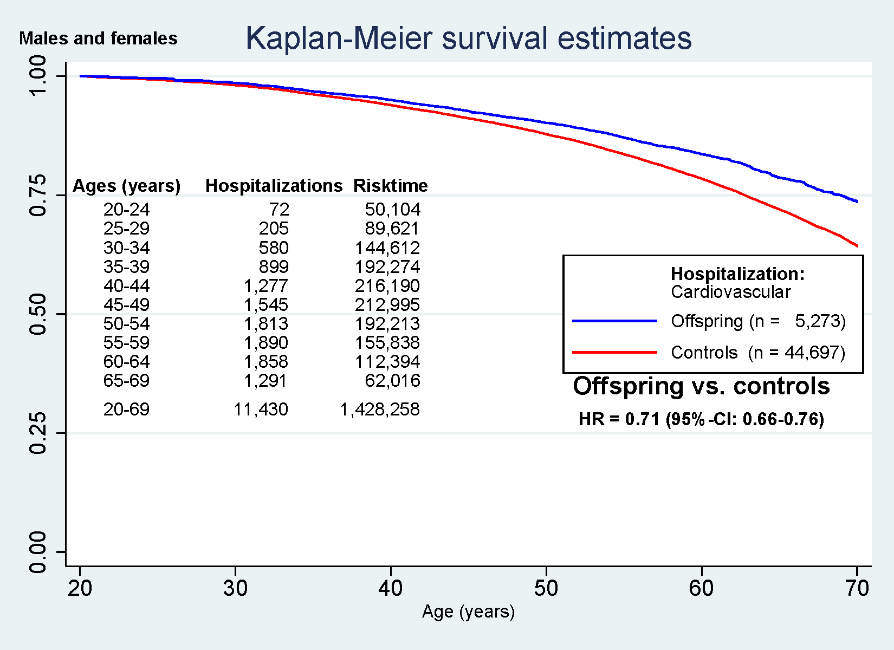


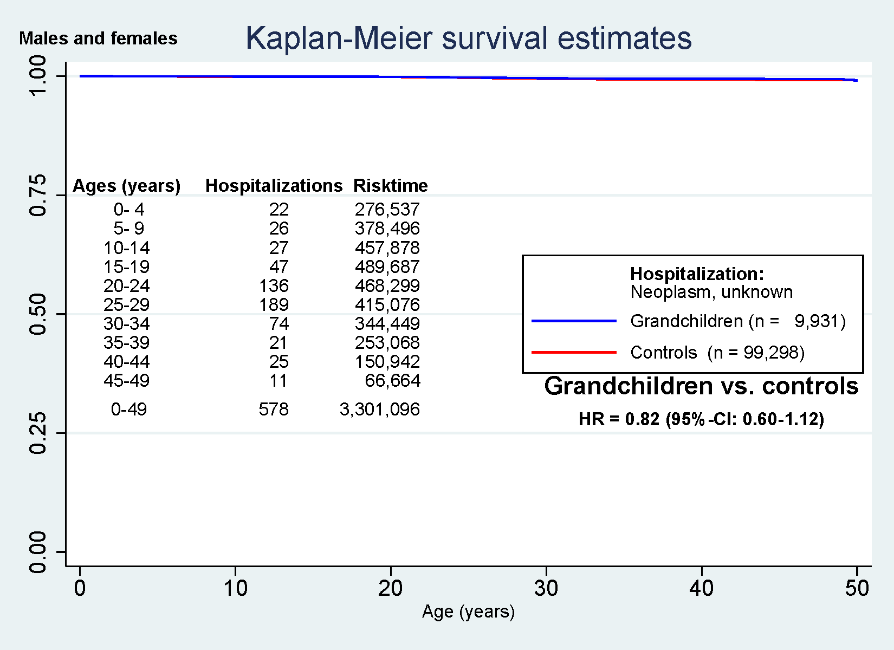

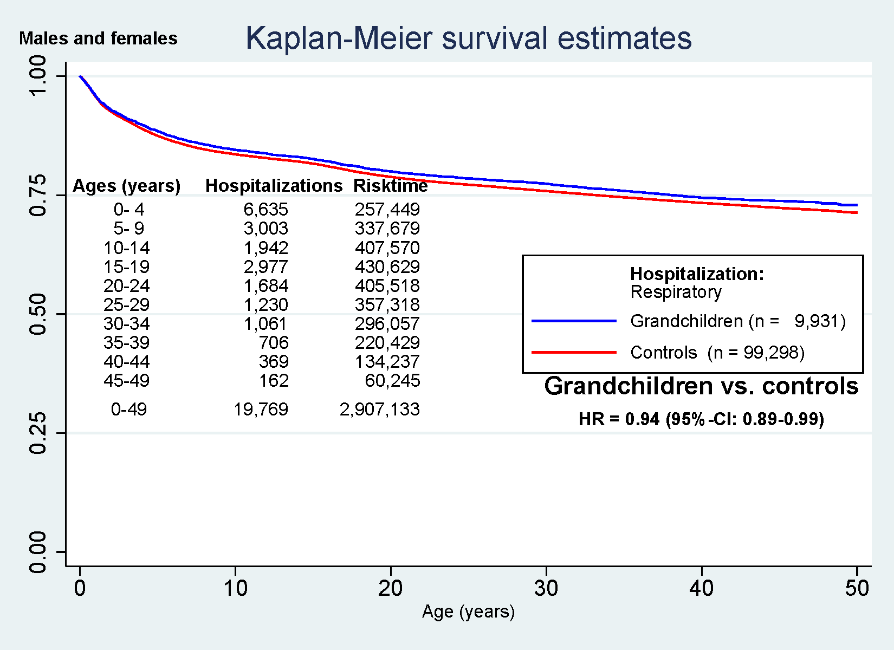


**Figure S4**. Kaplan-Meier survival curve estimates, and age distribution of outcomes and individuals at risk among offspring vs. controls and grandchildren vs. controls for two low-incidence diseases (offspring: anemia; grandchildren: neoplasms, unknown/uncertain) and two high-incidence diseases (offspring: cardiovascular; grandchildren: respiratory)


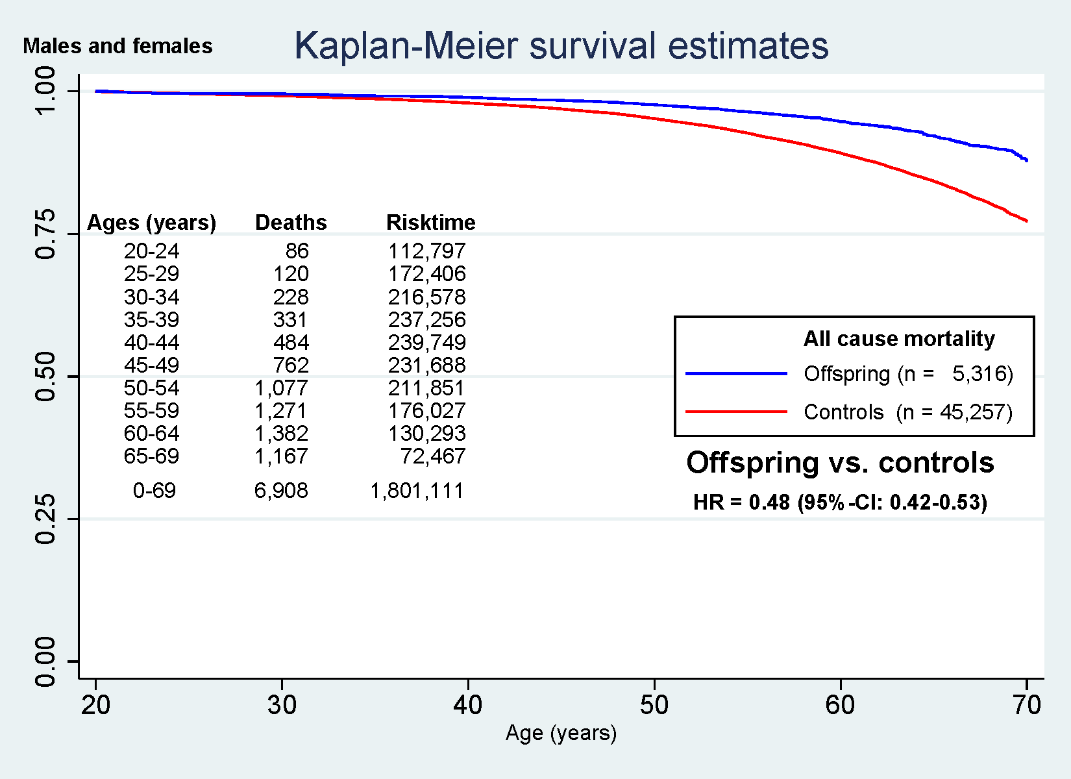

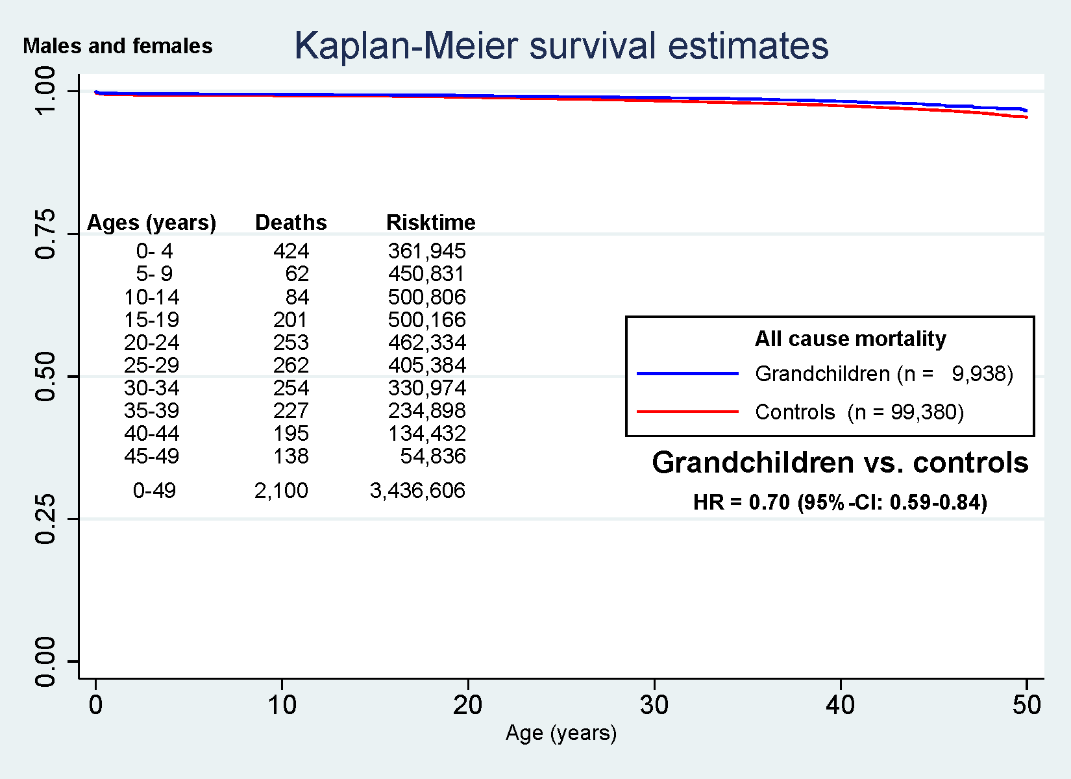


**Figure S5**. Kaplan-Meier survival curve estimates, and age distribution of outcomes and individuals at risk, among offspring vs controls and grandchildren vs. controls for all-cause mortality.


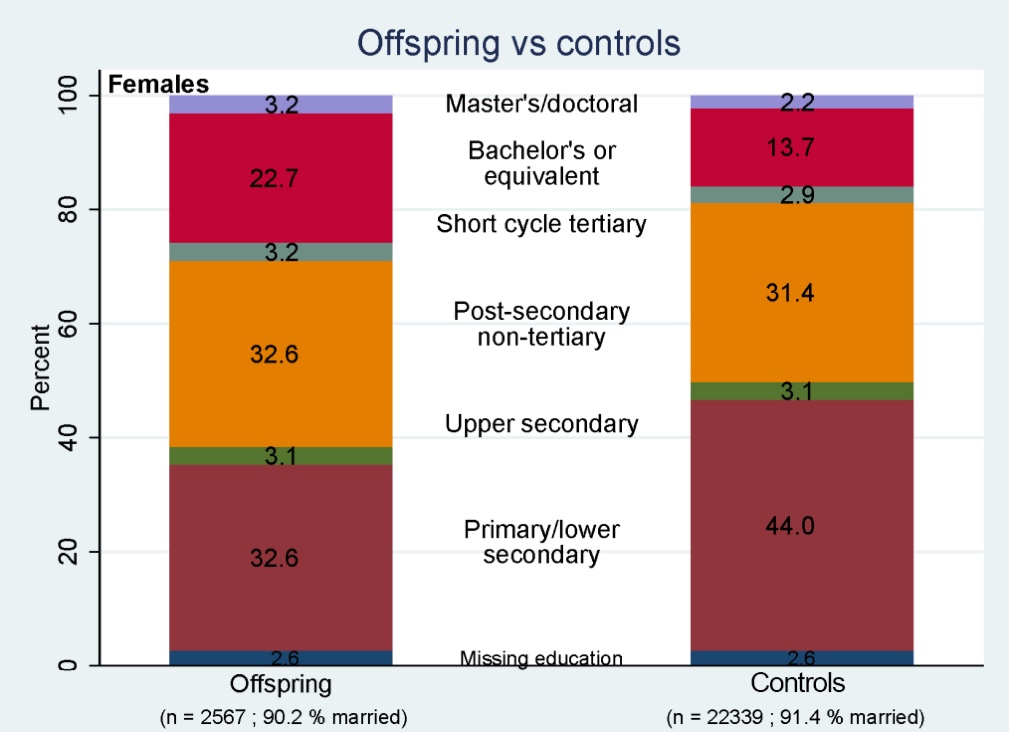

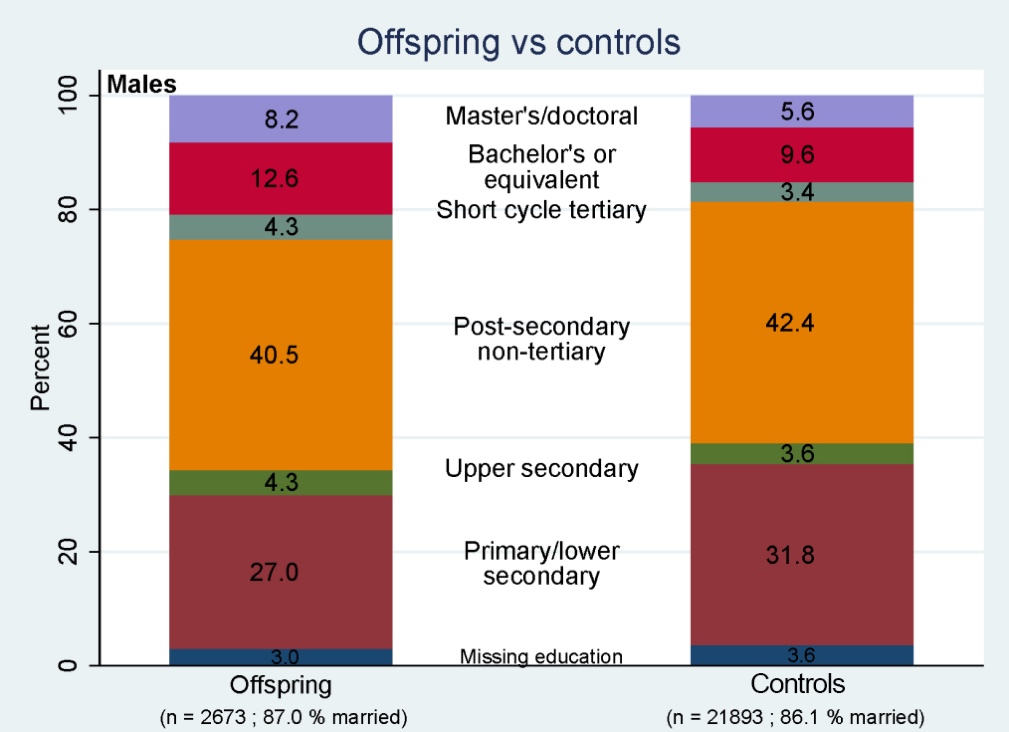


**Figure S6**. Offspring of Danish longevity-enriched families vs. age and sex-matched controls – proportions with highest attained educational level at age 30 in % in each of the categories *missing*, *primary and lower secondary*, *upper secondary*, *post-secondary non-tertiary*, *short cycle tertiary*, *Bachelor’s or equivalent*, or *Masters’/Doctoral or equivalent* (males: upper panel; females: lower panel)


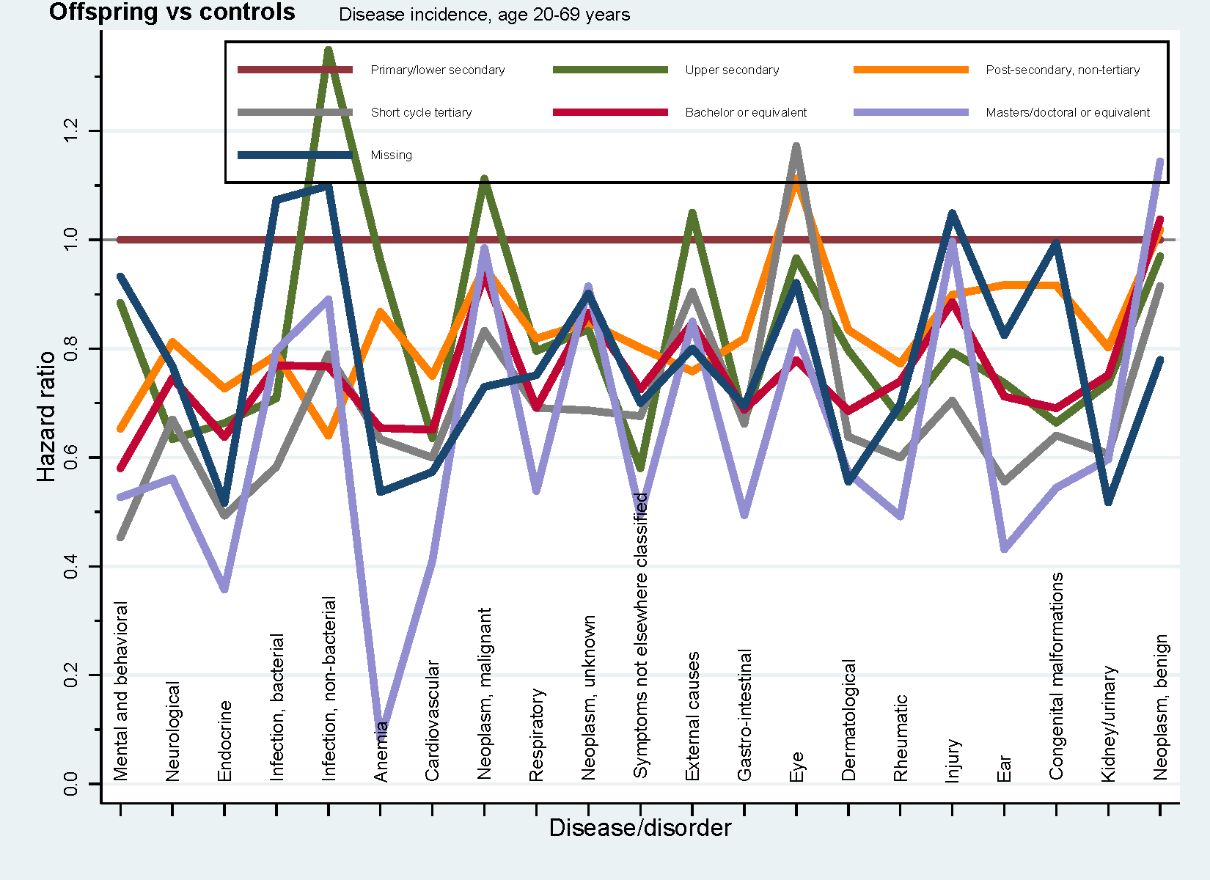


**Figure S7**. Offspring of Danish longevity-enriched families vs. age and sex-matched controls - association of highest attained educational level at age 30 with disease incidence for each of the 22 disease categories using the *primary and lower secondary education* category as reference (maroon curve, all HRs are identically equal to 1, and with six contrasting educational categories: *upper secondary education* (forest green), *post-secondary non-tertiary education* (orange), *short cycle* *tertiary education* (gray), *Bachelor’s or equivalent level* (cranberry), *Masters’/Doctoral or equivalent level* (lavender) or *missing educational level* (dark navy).


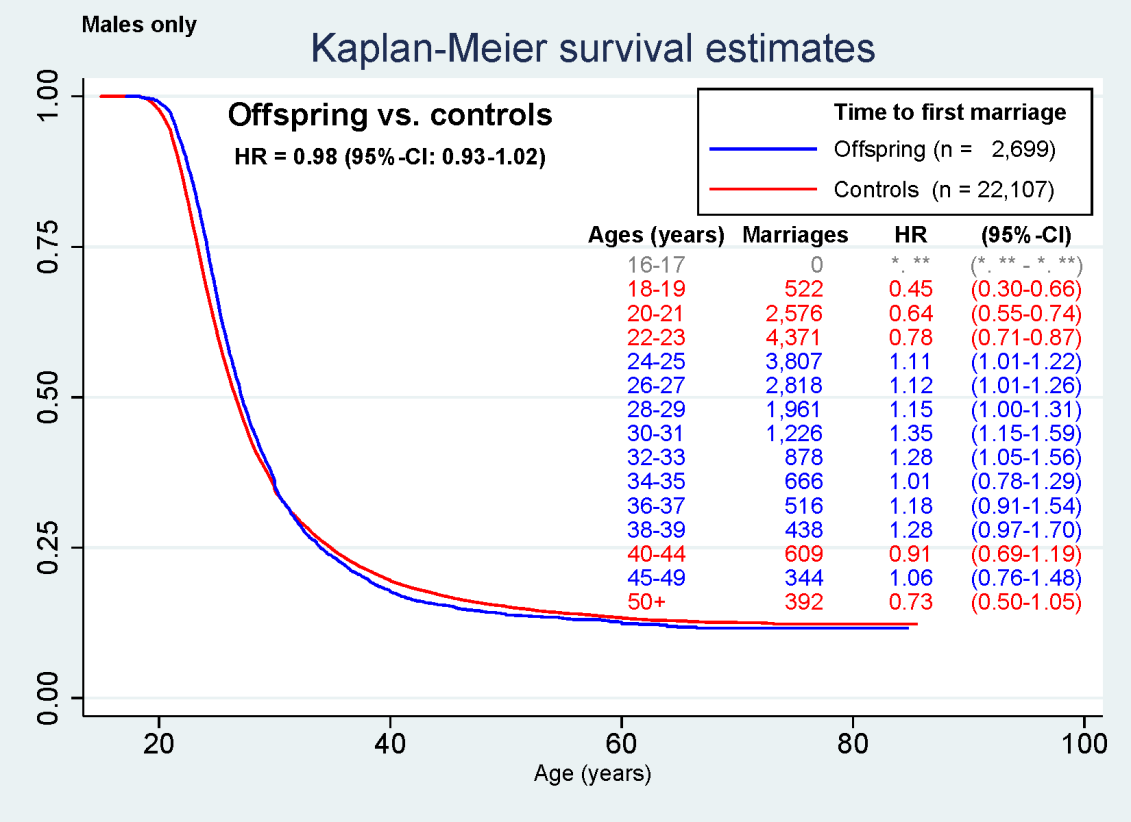

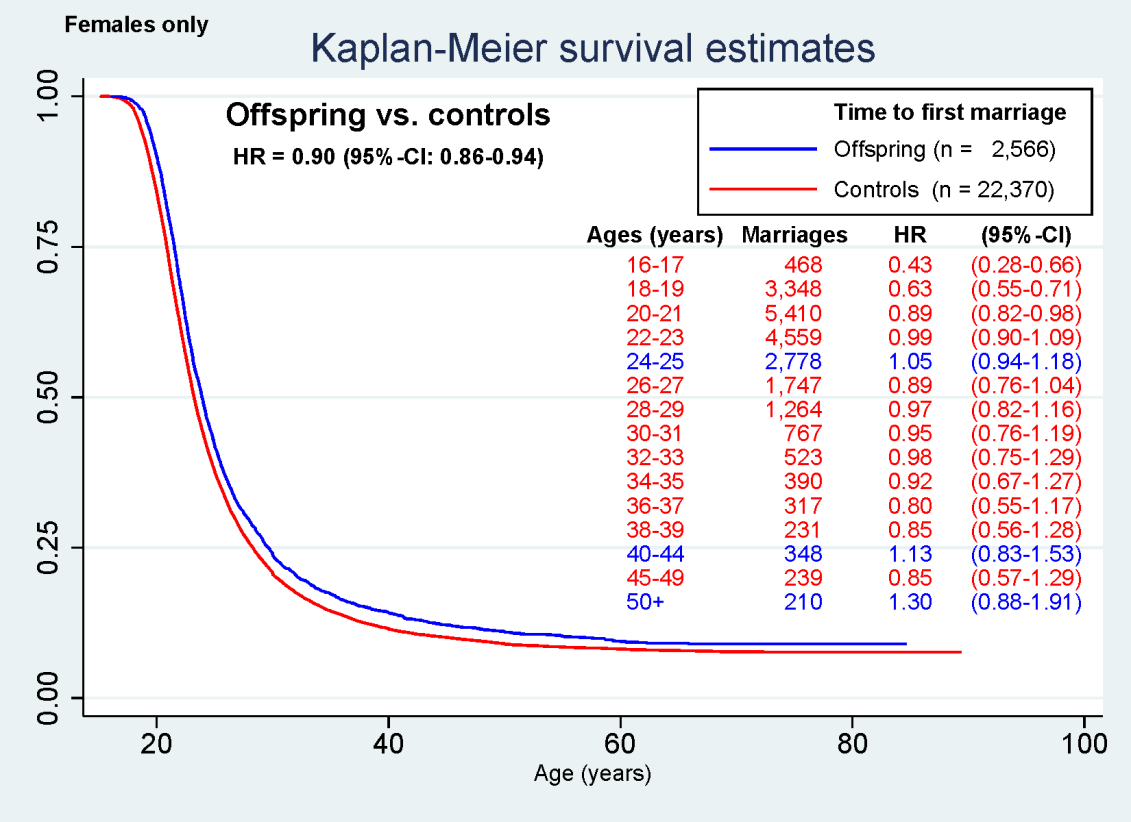


**Figure S8**. Kaplan-Meier survival curves of time to first marriage on LEF offspring and controls and age-specific hazard ratios (upper panel: males; lower panel: females)


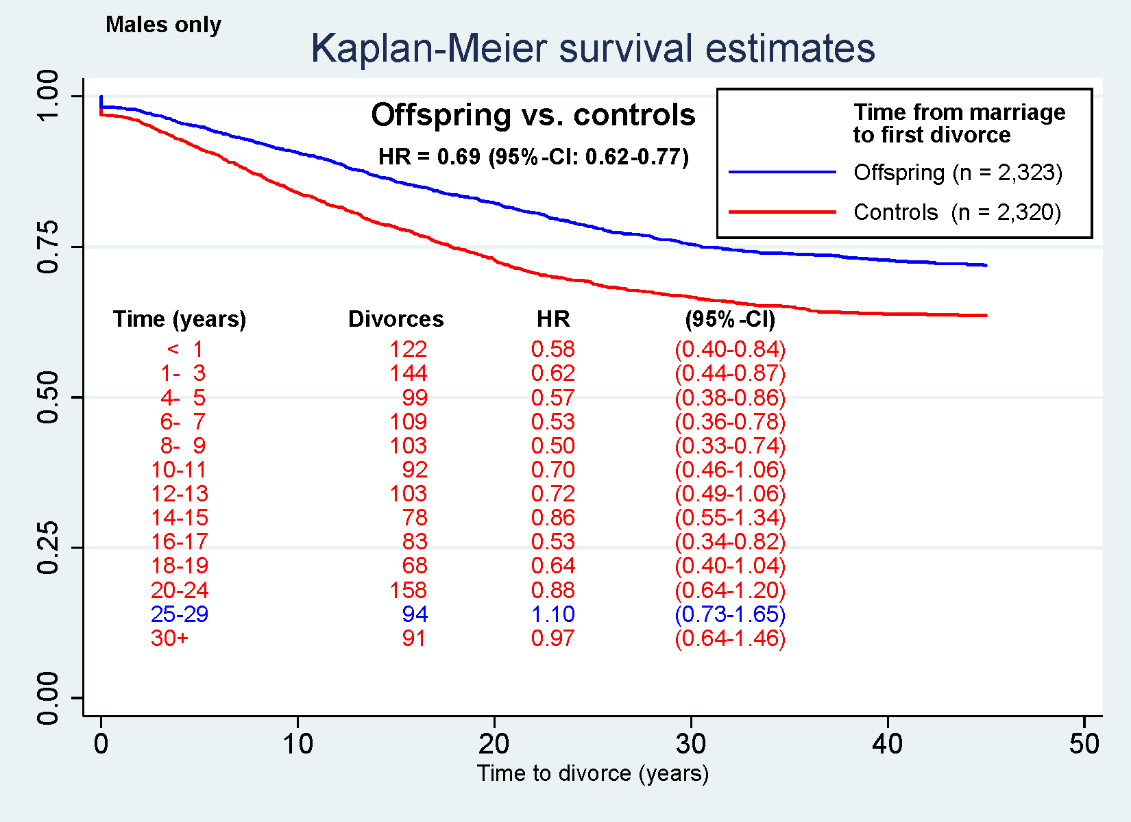

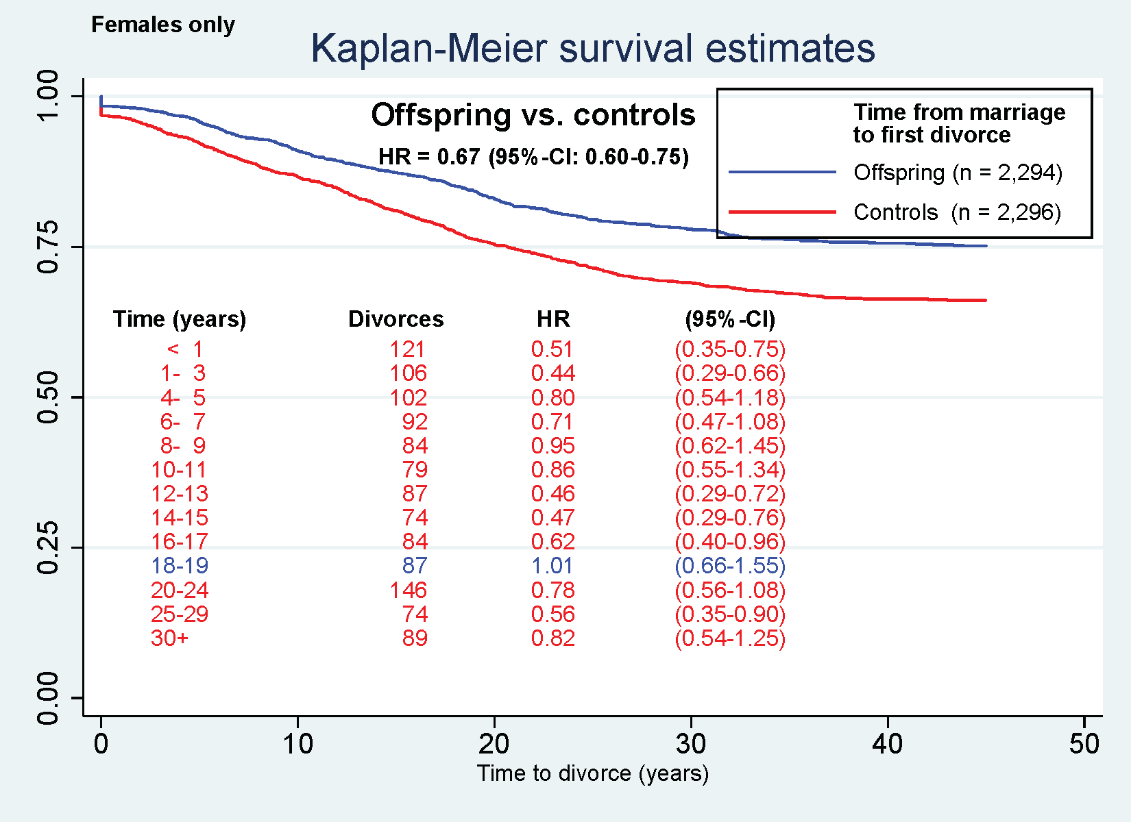


**Figure S9**. Kaplan-Meier survival curves of time from first marriage to first divorce among Danish LEF offspring and controls and age-specific hazard ratios (upper panel: males; lower panel: females)


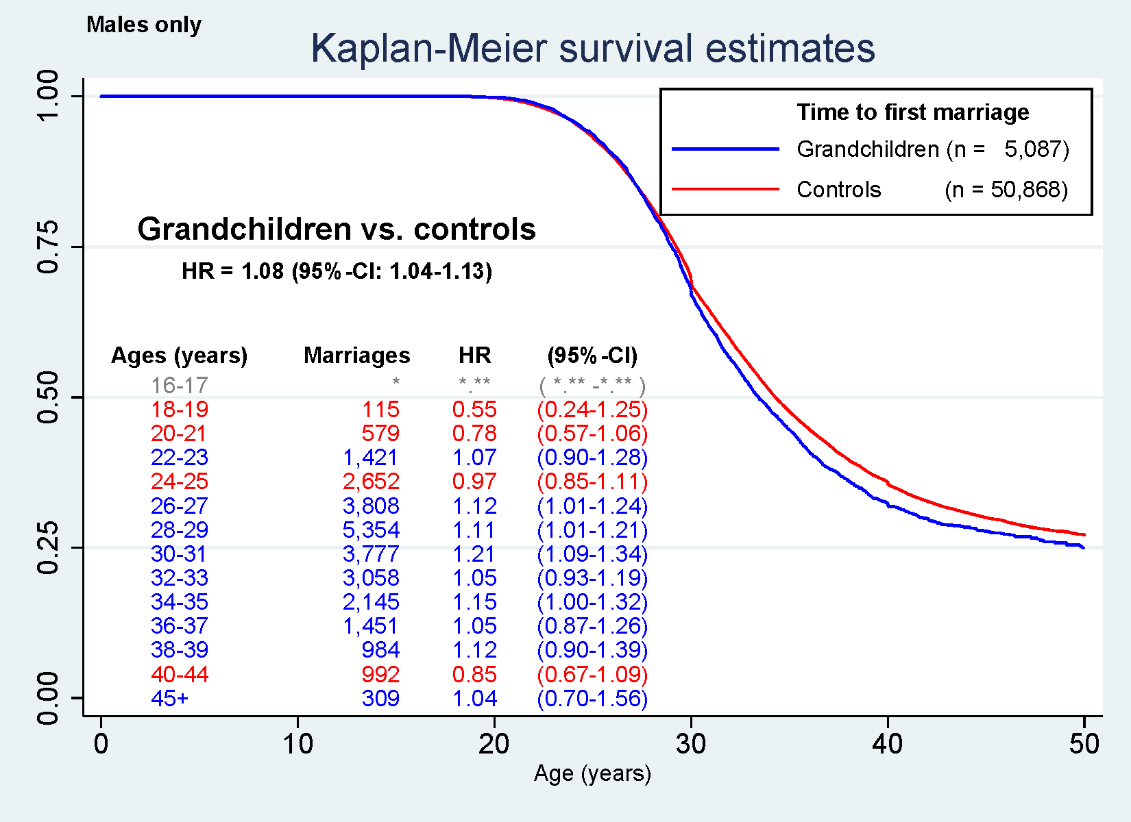

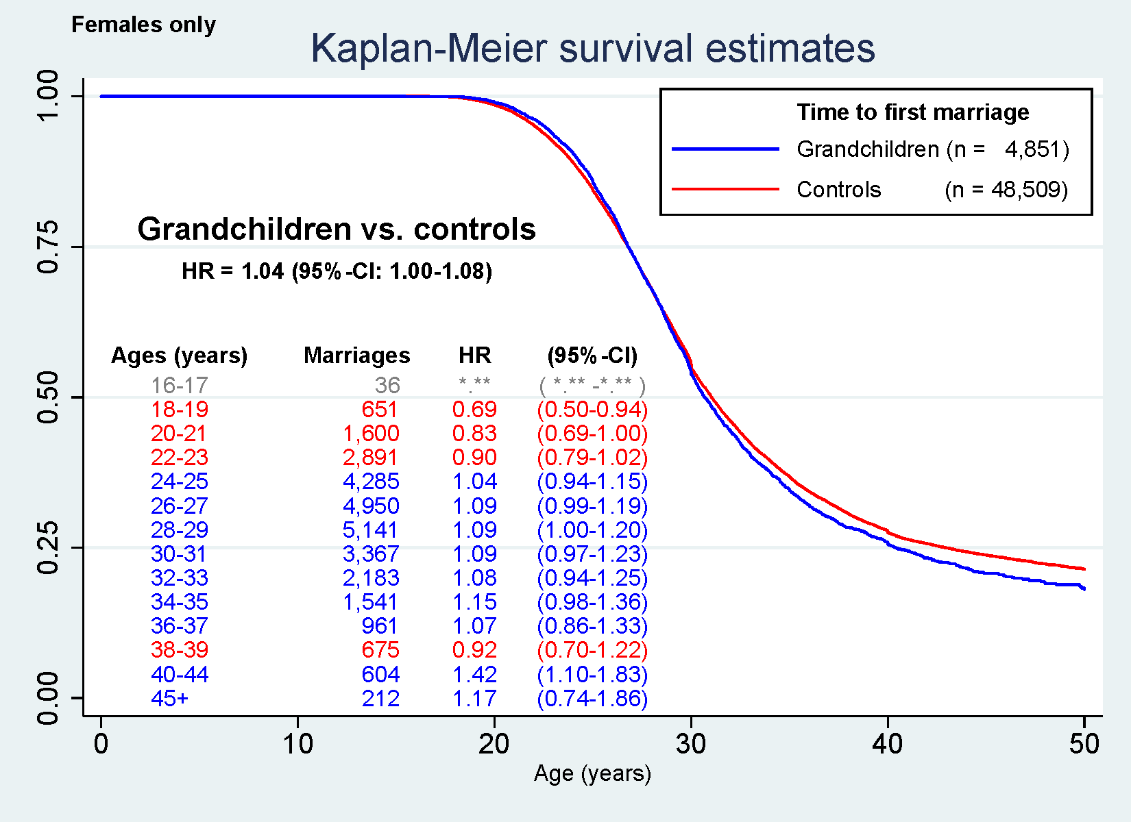


**Figure S10**. Kaplan-Meier survival curves of time to first marriage among Danish LEF grandchildren and controls and age-specific hazard ratios (upper panel: males; lower panel: females)


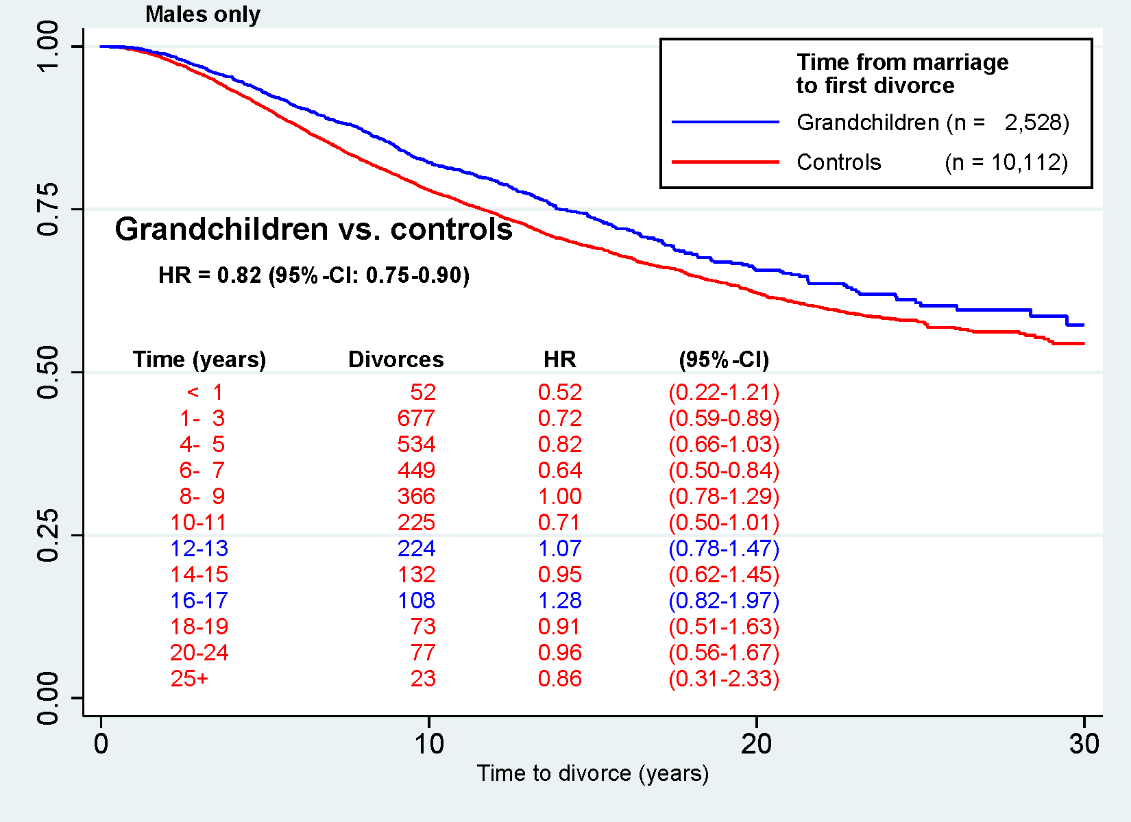

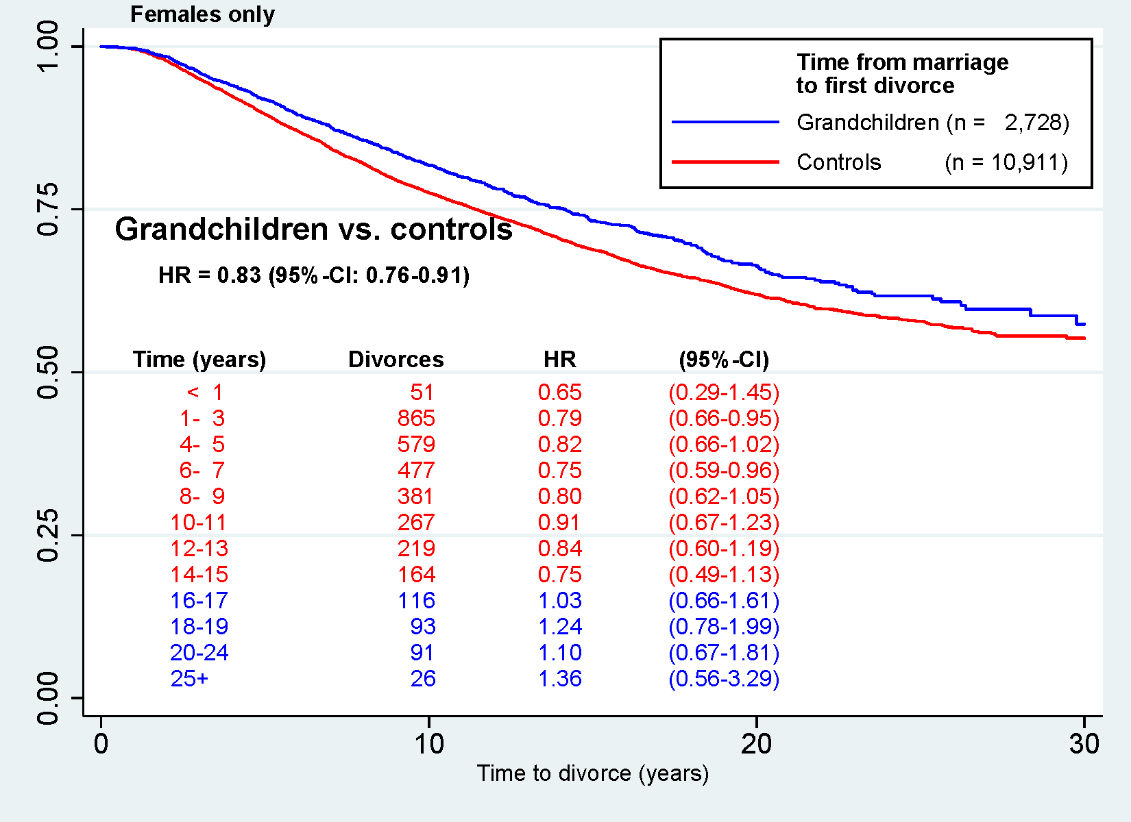


**Figure S11**. Kaplan-Meier survival curves of time from first marriage to first divorce among Danish LEF grandchildren and controls and age-specific hazard ratios (upper panel: males; lower panel: females)


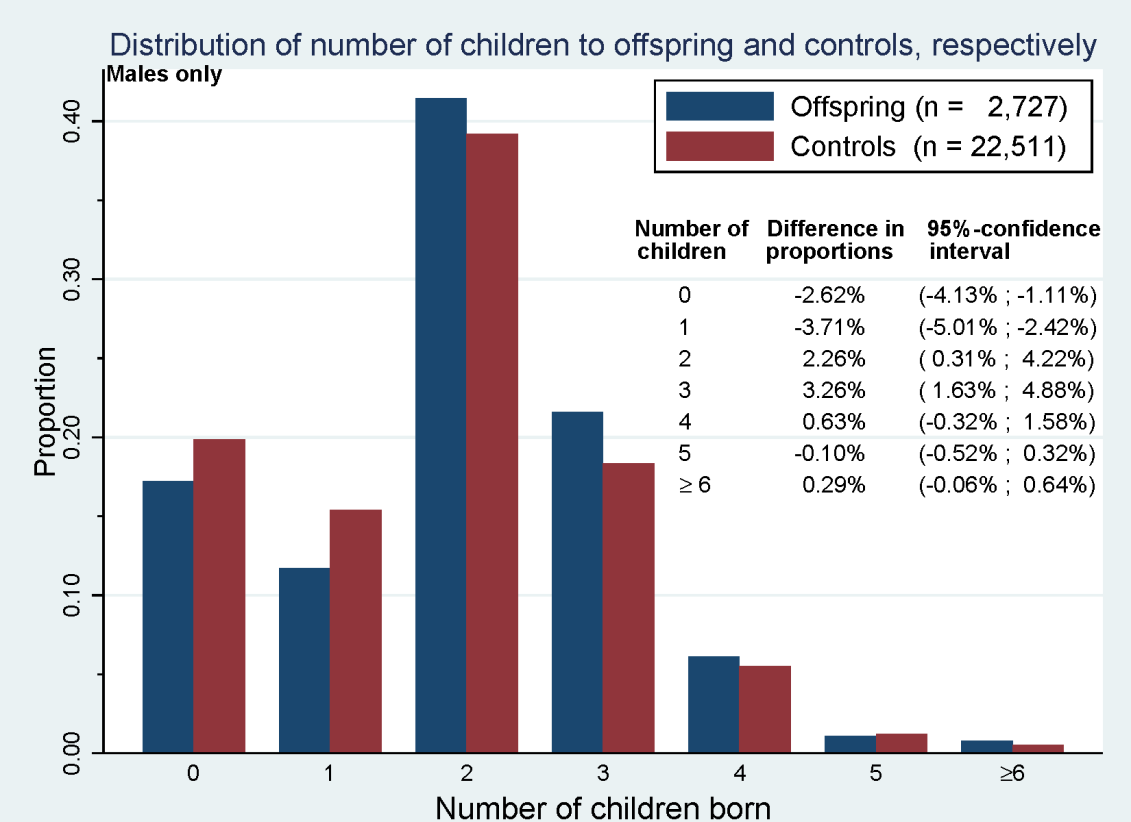

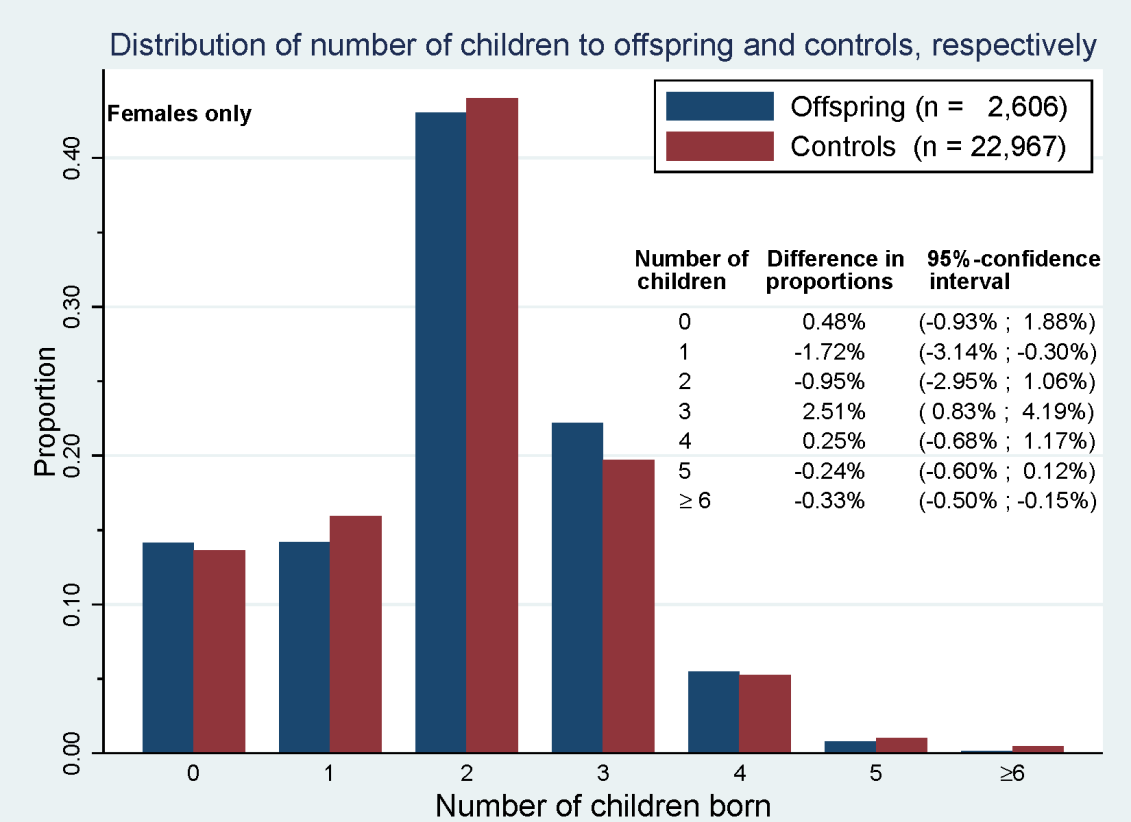


**Figure S12**. Distribution of number of children born to Danish LEF offspring and controls as well as differences (offspring-controls) in proportions (upper panel: males; lower panel: females)


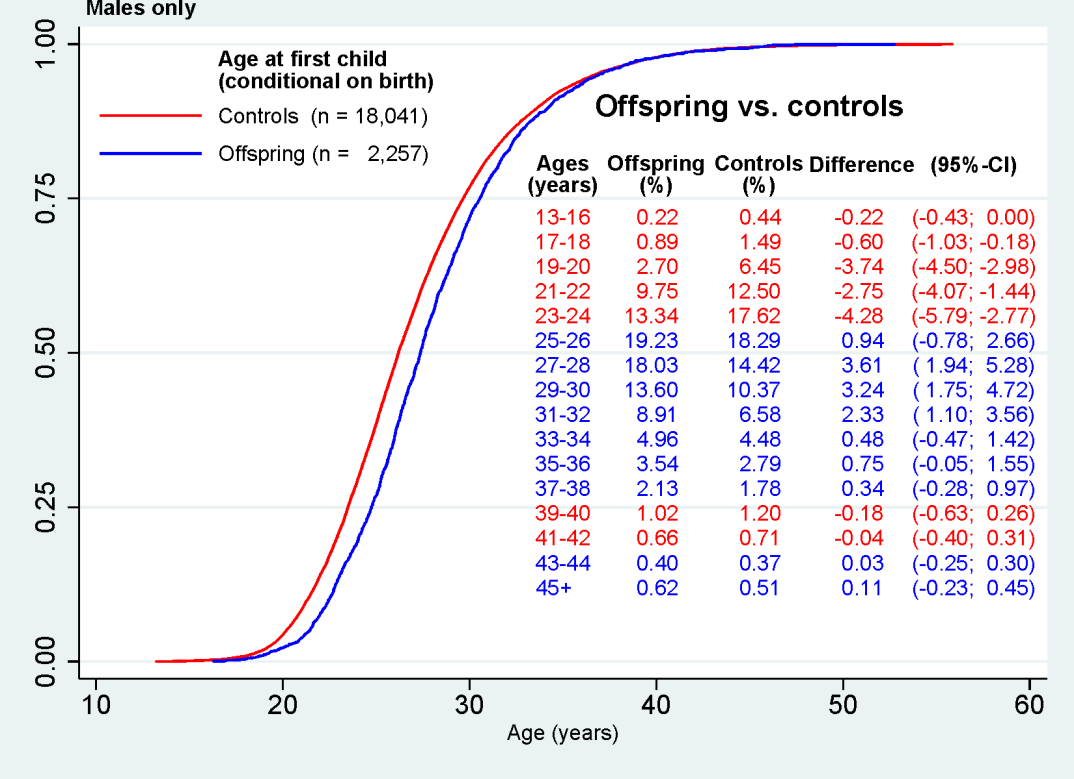

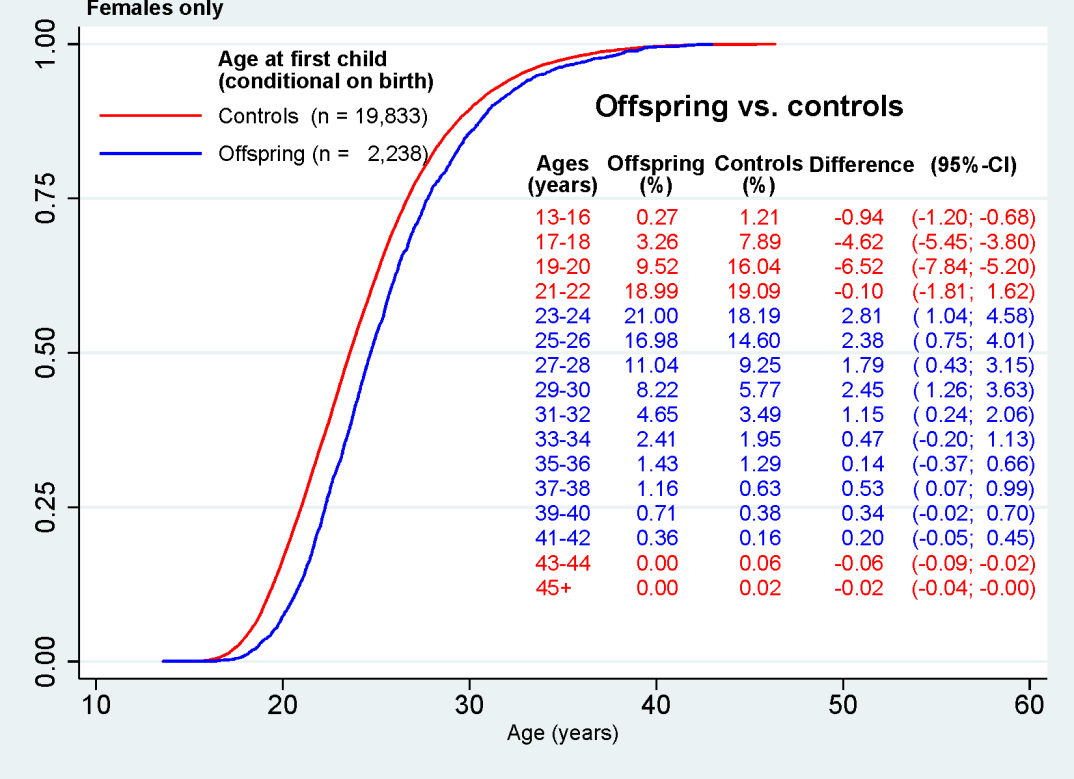


**Figure S13.** Distribution function and interval densities of age at first child among offspring of Danish longevity-enriched families and controls, conditional on giving birth and stratified on sex (upper panel: males; lower panel: females).


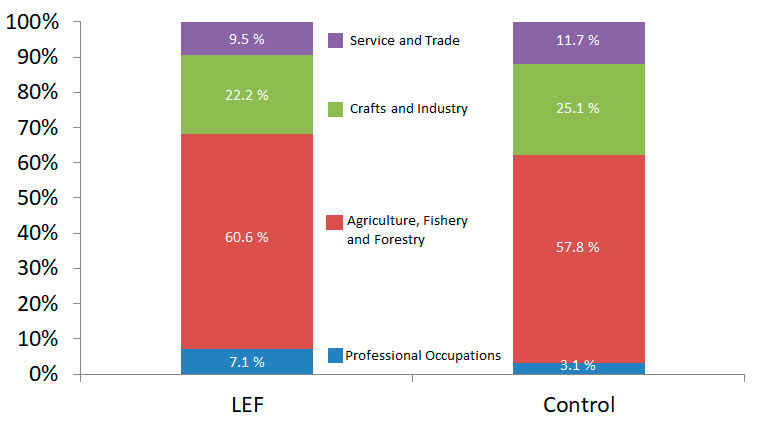


**Figure S14**. Occupation distribution in the 1916 Census for the G0 generation of 641 Longevity Enriched Families (4 missing) and 350 Control families (8 missing).
